# Regulation of alternative splicing by the circadian clock and food related cues

**DOI:** 10.1186/gb-2012-13-6-r54

**Published:** 2012-06-21

**Authors:** Nicholas J McGlincy, Amandine Valomon, Johanna E Chesham, Elizabeth S Maywood, Michael H Hastings, Jernej Ule

**Affiliations:** 1 MRC Laboratory of Molecular Biology, Hills Road, Cambridge, CB2 0QH, UK; 2 Institute of Pharmacology and Toxicology, University of Zurich, Winterthurerstrasse 190, CH-8057 Zürich, Switzerland

## Abstract

**Background:**

The circadian clock orchestrates daily rhythms in metabolism, physiology and behaviour that allow organisms to anticipate regular changes in their environment, increasing their adaptation. Such circadian phenotypes are underpinned by daily rhythms in gene expression. Little is known, however, about the contribution of post-transcriptional processes, particularly alternative splicing.

**Results:**

Using Affymetrix mouse exon-arrays, we identified exons with circadian alternative splicing in the liver. Validated circadian exons were regulated in a tissue-dependent manner and were present in genes with circadian transcript abundance. Furthermore, an analysis of circadian mutant *Vipr2^-/- ^*mice revealed the existence of distinct physiological pathways controlling circadian alternative splicing and RNA binding protein expression, with contrasting dependence on Vipr2-mediated physiological signals. This view was corroborated by the analysis of the effect of fasting on circadian alternative splicing. Feeding is an important circadian stimulus, and we found that fasting both modulates hepatic circadian alternative splicing in an exon-dependent manner and changes the temporal relationship with transcript-level expression.

**Conclusions:**

The circadian clock regulates alternative splicing in a manner that is both tissue-dependent and concurrent with circadian transcript abundance. This adds a novel temporal dimension to the regulation of mammalian alternative splicing. Moreover, our results demonstrate that circadian alternative splicing is regulated by the interaction between distinct physiological cues, and illustrates the capability of single genes to integrate circadian signals at different levels of regulation.

## Background

Alternative splicing is of particular interest amongst the post-transcriptional processes that regulate gene expression. Through effecting the production of mRNA isoforms with different exonic composition from a single gene, alternative splicing significantly increases the functional and regulatory diversity encoded by the genome. The *Drosophila *gene *Dscam *exemplifies this. *Dscam *contains 95 alternative cassette exons, corresponding to different portions of the Dscam receptor. Through combinatorial use of these exons, the *Dscam *gene has the potential to encode 38,016 distinct isoforms [1]. This vast isoform diversity provides the molecular basis for neurite self-avoidance during the development of the *Drosophila *nervous system [1].

Alternative splicing is particularly widespread in mammalian genes, affecting approximately 95% and 80% of multi-exon genes in *Homo sapiens *and *Mus musculus*, respectively [2,3]. Moreover, alternative splicing is highly regulated, with global patterns of exon inclusion reflecting different biological settings. These patterns are regulated by the abundance, activity and binding position of various auxiliary splicing factors (for example, SR (serine-arginine) and heterogenous nuclear ribonucleoprotein (hnRNP) families of proteins), the transcriptional elongation kinetics and potentially chromatin modifications [4-6]. Classically, alternative splicing patterns and their regulation have been studied based on the differences associated with the development of different tissues, or with disease states [7,8]. Indeed, what we currently describe as tissue-specific splicing likely reflects an average of the different cell-type-specific splicing patterns within an organ or tissue. This has been elegantly illustrated by the recent description of layer-specific alternative splicing events within the mouse somatosensory cortex [9]. More recently, the study of alternative splicing regulation has been extended to responses to the environment, either acute, such as the depolarization of cultured neurons [10], or chronic, such as the long-term nutritional state of the organism [11]. Furthermore, links have been identified between the cell cycle and alternative splicing [12]. However, an aspect of alternative splicing that is yet to be considered in mammals is its regulation by physiological systems such as the circadian clock.

The circadian clock is a cell autonomous biological oscillator that drives daily (that is, circadian) rhythms in behaviour, metabolism and physiology [13]. These circadian rhythms are a pervasive influence on biology, being evident in organisms ranging in complexity from bacteria to mammals [14]. They confer a selective advantage on organisms by allowing them to anticipate regular changes in their environment and to temporally segregate incompatible metabolic processes [15,16]. Indeed, their importance for humans can be illustrated by the long-term negative health impact of behaviours that require prolonged nocturnal activity, such as rotational shift-work and trans-meridian travel (for example, increased risk of colorectal, endometrial and prostate cancer) [17], and the association of circadian dysfunction with metabolic syndrome and major neurological and psychological disorders (for example, Alzheimer's, Parkinson's, unipolar and bipolar depression and autism spectrum disorders) [18].

Circadian rhythms are underpinned by daily rhythms of gene expression. The transcriptional component of these rhythms is comparatively well understood. The cell-autonomous clock may be considered to comprise a number of interlocking transcriptional negative feedback loops [19]. The circadian variation in abundance of the positive (Clock and Arntl (Bmal1)) and negative (Per1 and Per2 and Cry1 and Cry2) components of these loops in turn drive the circadian transcription of both direct targets genome-wide (including themselves) and a cascade of circadian output transcription factors, which together mediate the circadian transcriptional profile of a cell type or tissue [13,20]. Little is known, however, of the contribution of post-transcriptional processes to circadian rhythms, particularly in mammals [21,22]. Nevertheless, complementary studies of the murine circadian transcriptome and proteome, in both liver and the suprachiasmatic nucleus (SCN) of the hypothalamus, have revealed a frequent lack of concordance between protein and mRNA rhythms [23,24], highlighting the importance of post-transcriptional control in regulating clock outputs. Moreover, recent studies in *Drosophila melanogaster *have also shown the importance of post-transcriptional processes for the proper functioning of the circadian clock itself, particularly the microRNA *bantam *[25] and the translational regulator *twenty-four *[26].

In the present study, we have determined that the circadian clock regulates alternative splicing in the mouse and have examined how this is controlled by the different physiological pathways that orchestrate circadian information in a whole animal. We have found that circadian alternative splicing is tissue-dependent in both phase and amplitude, and that it is frequently present in genes with circadian transcript abundance. Feeding is an important circadian stimulus for peripheral organs, and we found that not only the temporal pattern of examined circadian exons, but also their temporal relationship to transcript-level expression, were modulated in the liver by fasting conditions. Moreover, analysis of circadian mutant *Vipr2^-/- ^*mice showed that these exons were under the control of the local liver clock, but that many identified circadian splicing factors were not. Taken together, our data indicate that circadian alternative splicing integrates circadian information from multiple physiological sources and provide insights into the candidate regulators of circadian splicing.

## Results

### The circadian clock regulates alternative splicing in a tissue-dependent manner

**Figure 1:**
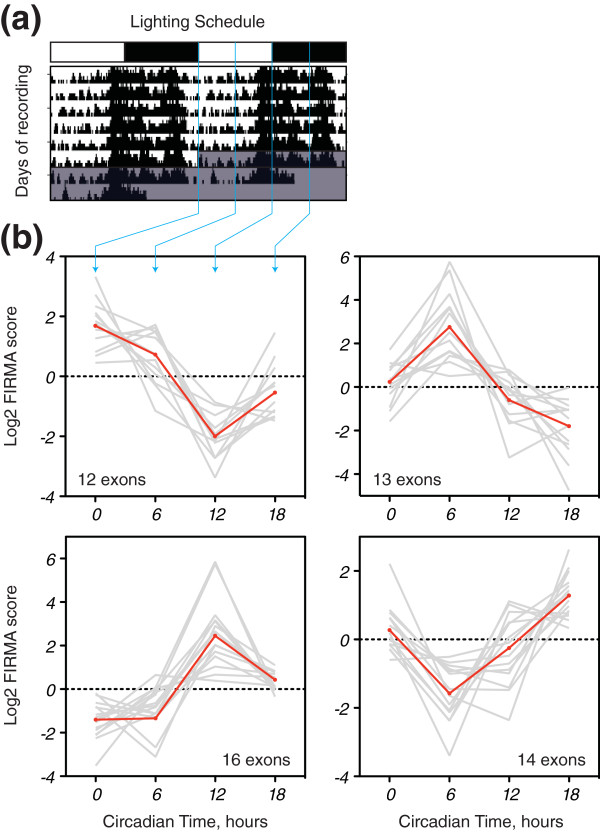
**FIRMA analysis of exon-arrays reveals circadian alternative splicing in mouse liver**. **(a) **Representative double-plotted actogram showing the circadian locomotor activity of the wild-type mice used in this study. Vertical black lines represent infrared beam brakes, which reflect locomotor activity. LD, 12 h light followed by 12 h dark-red light; DD, constant dark-red light. The white and black boxes atop the actogram represent the lighting schedule (LD)/subjective day and night (DD). Descending blue lines indicate the times at which samples were harvested. **(b) **Circadian FIRMA score profiles were clustered into four groups using a self-organizing tree algorithm [78]. For each panel, grey lines represent the exon-probesets comprising that cluster, whereas the red line represents the cluster centroid. Cluster 1, top left; cluster 2, top right; cluster 3, bottom left; cluster 4, bottom right (Additional file 2).

We sought to identify circadian regulation of alternative splicing in mouse liver. The circadian phenotype of liver provides a paradigm for both the circadian control of metabolism and the regulation of a peripheral oscillator by the master-clock in the SCN of the hypothalamus [13]. Thus, wild-type male mice were kept under 12 h:12 h light:dim red light (LD), in which they displayed the expected daily nocturnal pattern of locomotor activity (Figure 1a). This pattern continued when the animals were placed into constant dim red light (DD; Figure 1a), confirming their circadian behaviour. In the second day of DD, animals were harvested at circadian time (CT) 0, 6, 12 and 18 and their livers extracted for genome-wide analysis of gene expression and alternative splicing using the Affymetrix exon-array. The exon-arrays faithfully reproduced the temporal expression patterns of the canonical circadian genes *Dbp *and *Arntl *(*Bmal1*), as measured by quantitative PCR (QPCR; Figure S1 in Additional file 1), confirming these samples as rhythmic at the molecular level.

In order to identify circadian alternative exons, we analyzed the exon-array data using FIRMA (Finding Isoforms using Robust Multichip Analysis). FIRMA predicts alternative splicing in a single sample as a departure from the transcript-level expression estimate produced by the Robust Multichip Analysis (RMA) model [27]. Thus, for each exon-probeset at each time point, a score is calculated that measures this departure (the FIRMA score), a score of 1 indicating no departure. FIRMA scores were then compared across circadian time by one-way ANOVA. Exon-probesets showing a significant effect of time (false discovery rate (FDR) corrected *P *< 0.05) were then screened to identify circadian patterns (see Materials and methods). This identified 55 exon-probesets from 47 genes with significant circadian variation in FIRMA score (Additional file 2). This corresponds to approximately 0.4% of the genes detectable on the array. Clustering of the circadian profiles of these exon-probesets revealed a variety of different phases (Figure 1b), suggesting that circadian regulation of alternative splicing occurs at all stages of the circadian cycle.

In order to examine the functions of a putative circadian splicing network, we sought to identify enriched functional annotations associated with the genes containing circadian exons. Despite the comparatively small number of genes examined, this revealed enrichments within the Kyoto Encyclopedia of Genes and Genomes (KEGG) pathways [28] representing the circadian clock itself, drug detoxification, caffeine and retinol metabolism and the peroxisome proliferator-activated receptor (PPAR) signaling pathway. These KEGG associations were supported by enrichments in Gene Ontology terms [29] and InterPRO domains [30] pertaining to the same processes, and the functions of the genes compromising them (Additional files 3, 4, and 5). Interestingly, hepatic drug metabolism was previously shown to be under circadian transcriptional control [31], suggesting that circadian phenotypes in peripheral organs are specified by control of gene expression at multiple levels.

**Figure 2:**
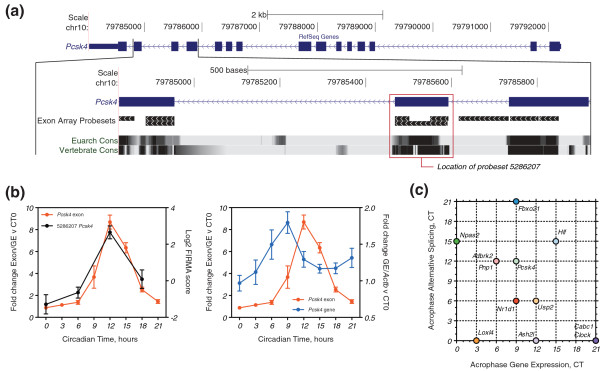
**Validated circadian exons show a variety of acrophases in alternative splicing and transcript-level expression**. **(a) **UCSC genome browser [73,79] screen-shots. Top: the structure of the Refseq mRNA and the location of the identified cassette exon for *Pcsk4 *(genome assembly mm9). Bottom: the closer genomic context of the identified exon, including the location of core exon-array probesets and phastCons conservation [80]. **(b) **Upper panel: plot of the log2 FIRMA score (black line, *n *= 3, standard error of the mean (SEM)) and alternative splicing (red line, *n *= 6, SEM) through time. Lower panel: plot of the alternative splicing (red line, *n *= 6, SEM) and transcript-level expression (blue line, *n *= 6, SEM) through time (GE, gene expression). **(c) **Scatter-plot relating the acrophase in transcript-level expression to the acrophase in alternative splicing for each of the validated exons. Acrophase is the circadian time of peak expression. Peak expression was defined as the circadian time of the largest positive fold change versus CT0. *Decr1 *is not included in this plot as its transcript-level expression was not rhythmic.

Circadian exons were validated in a separate animal experiment with greater temporal resolution (every 3 hours) and sensitivity (*n *= 6). The proper rhythmic expression of *Dbp*, *Per2 *and *Arntl *was confirmed in liver, lung and kidney (Figure S2 in Additional file 1). Validation was performed by exon-specific QPCR normalized to gene expression. We selected 25 exon-probesets from across the four FIRMA score phase clusters (Figure 1b). Of these 25, 13 were confirmed as rhythmic in liver (*P *< 0.05, one-way ANOVA for effect of time; Figure 2; Figure S3 in Additional file 1). These exons comprised nine cassette exons (*Pcsk4*, *Npas2*, *Usp2*, *Fbxo21*, *Nr1d1*, *Ash2l*, *Loxl4*, *Adrbk2 *and *Clock*), two alternative initial exons (*Hlf *and *Cabc1*) and two alternative polyadenylation sites (*Pnp1 *and *Decr1*). In 10 of 13 cases, the FIRMA score correlated either positively or negatively with the exon-specific QPCR results (*r^2 ^*≥ 0.6).

Tissue-dependent alternative splicing is most often regulated independently of transcript-level expression, such that tissue-specific exons are rarely present in genes with transcript-level regulation in the same tissue [7]. In contrast, the vast majority of our validated exons were present in genes with transcript-level circadian regulation in liver (Figure 2; Figure S3 in Additional file 1). For example, *Pcsk4 *circadian alternative splicing displayed a large peak at CT12, and its temporal pattern was highly concordant with that of the FIRMA score (Pearon's *r *= 0.99; Figure 2b, left panel). Strikingly, *Pcsk4 *transcript-level expression was also rhythmic, but with a different phase (peaking at CT9) and amplitude (Figure 2b, right panel). The exception was *Decr1*, where the exon-specific expression (in this case, an alternative portion of the 3' UTR) was rhythmic, but the transcript-level expression was not (Figure S3j in Additional file 1). In spite of the evident co-regulation of circadian alternative splicing and transcript-level expression, we could observe no systematic relationship between the acrophases (circadian time of peak expression) of alternative splicing and transcript-level changes across all exons (Figure 2c). Thus, it would appear that these two modes of gene expression are regulated independently in our validated exons.

**Figure 3:**
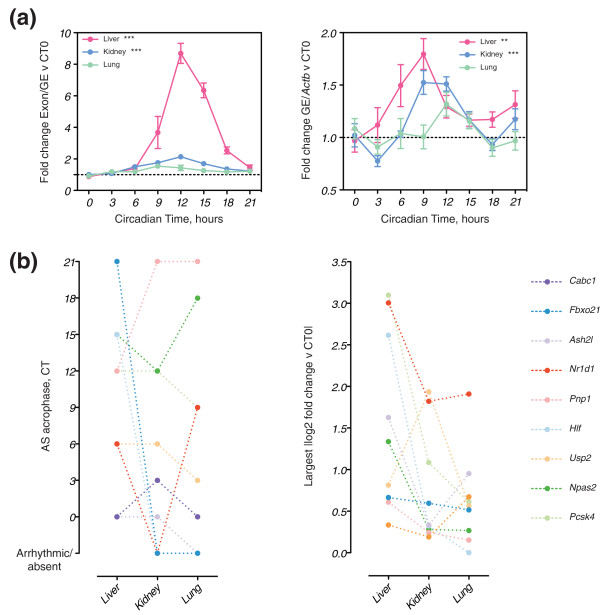
**The amplitude and phase of circadian exons is tissue-dependent**. **(a) **Plots of *Pcsk4 *alternative splicing (left panel) and transcript-level expression (right panel, GE, gene expression) through time in liver (pink), kidney (blue) and lung (green). In each case, *n *= 6 and standard error of the mean. Asterisks indicate results of a one-way ANOVA for an effect of time (**P ≤ 0.01, ***P ≤ 0.001). Individual tissue time-courses were examined where a two-way ANOVA had revealed a significant (*P *≤ 0.05) main effect of tissue and/or an interaction effect between tissue and time. **(b) **Summary of alternative splicing data from the other validated exons (Figure S4 in Additional file 1). Left panel: acrophase of alternative splicing (AS) in three tissues. Acrophase was defined as the circadian time of the largest positive fold change versus CT0. Right panel: amplitude of alternative splicing in three tissues. Amplitude was defined as the biggest absolute value of fold change versus CT0 for exon-specific QPCR.

The regulation of alternative splicing is frequently tissue-dependent [2]. Moreover, the same is also true for circadian transcription [32]. To examine the tissue dependence of circadian alternative splicing, we analyzed some of our validated exons in lung and kidney (Figure 3; Figure S4 in Additional file 1). An example of the effects observed is illustrated with *Pcsk4 *(Figure 3a). The amplitude of the rhythm in alternative splicing observed in the liver was much greater than that observed in the kidney, while alternative splicing in the lung was essentially arrhythmic (Figure 3a, left panel). Indeed, seven out of nine exons showed a splicing change of greater amplitude in the liver compared to the lung and kidney (Figure 3b, right panel). Furthermore, the decrease in amplitude of alternative splicing from liver to kidney was not associated with an equivalent decrease in the amplitude of the rhythm in the transcript-level expression, while, similarly to the alternative splicing, transcript-level expression in the lung was also essentially arrhythmic (Figure 3a, right panel). Strikingly, the acrophases of the splicing changes were also frequently tissue-dependent (Figure 3b, left panel). All events showed a tissue-dependent difference in splicing acrophase. Two of the nine events (*Hlf *and *Fbxo21*) were entirely liver specific, being arrhythmic or entirely absent from lung and kidney. Of the remaining seven, three were different in both tissues compared to liver, whereas four were only different in one of the two tissues (Figure 3b, left panel). For these seven exons the individual time-differences in acrophase between liver and each of lung and kidney were examined. Seven time-differences were of 3 hours and two were of 9 hours, while four had no difference in acrophase. Taken together, these data indicate that both the acrophase and amplitude of circadian alternative splicing is tissue-dependent.

The tissue-dependence of the temporal relationship between alternative splicing and transcript-level changes grouped our validated exons into two categories. For seven out of nine exons, this temporal relationship was not consistent across tissues. Either the transcript-level regulation was present in all tissues and circadian alternative splicing was present predominantly in liver - for example, *Npas2 *(Figure S4 in Additional file 1h) - or a variety of patterns in both alternative splicing and transcript-level expression was observed - for example, *Nr1d1 *(Figure S4 in Additional file 1e) and *Cabc1 *(Figure S4 in Additional file 1g). By contrast, two out of nine exons had a temporal relationship between alternative splicing and transcript-level changes that was preserved across tissues. For example, *Usp2 *(Figure S4 in Additional file 1a) showed an approximate 6 hour delay between the acrophases of alternative splicing and transcript-level expression, which was preserved in kidney and lung. In conclusion, we have observed that the circadian regulation of alternative splicing is tissue-dependent, in terms of both phase and amplitude. Moreover, we have identified exons where the temporal relationship between alternative splicing and transcript-level expression appears to be preserved across tissues, suggesting that these two processes may be coupled in these particular cases. Thus, as is the case for circadian transcription, circadian regulation of alternative splicing adds an important temporal dimension to the gene expression profile of a tissue.

### Fasting modulates the temporal regulation of circadian exons, and their relationship with transcript-level expression

We sought to investigate the physiological pathways regulating circadian alternative splicing. The timing and availability of food can affect circadian patterns of mRNA abundance in the liver, including those of the core clock genes, independently of the master clock of the SCN [33-35]. This ability of the liver to be independently entrained by food illustrates the importance of circadian timing in the liver's role in the regulation of energy metabolism and xenobiotic detoxification [13]. In order to understand the relevance of this effect to the regulation of circadian alternative splicing, we examined the effect of fasting on the circadian alternative splicing of a number of our validated exons in liver.

**Figure 4:**
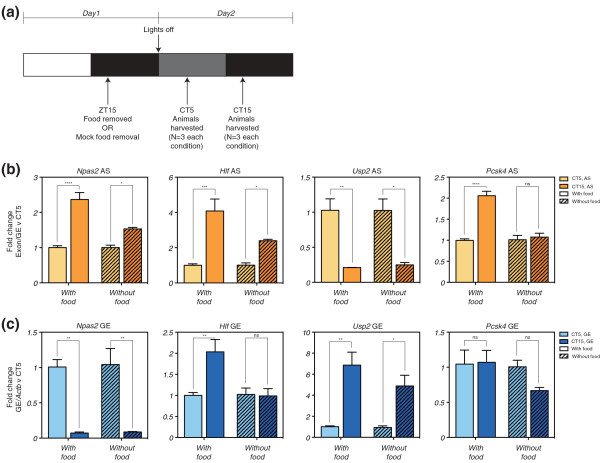
**Fasting modulates the temporal regulation of circadian exons, and their relationship with transcript-level expression**. **(a) **Schematic depicting the timing of important events during the two days of the fasting experiment; one day of LD followed by one day of DD. Colored boxes each depict 12 hour periods in the lighting schedule. White, day/lights on; black, night/dim red light; dark grey, subjective day/dim red light. ZT, Zeitgeber time (the time under an LD cycle); CT, circadian time (the corresponding time under a DD cycle). **(b) **Alternative splicing (AS) of four selected circadian exons. **(c) **Transcript-level expression for the same exons. For each combination of time point and feeding status, *n *= 3 and error bars represent standard error of the mean. The symbols above each pair of bars indicate the results of Bonferroni's *post hoc *test, comparing the two circadian time points in each feeding condition after a two-way ANOVA: Ns, not significant; *P ≤ 0.05; **P ≤ 0.01; ***P ≤ 0.001; ****P ≤ 0.0001.

Wild-type mice kept under LD were fed *ad libitum *until the night of day 1 of the experiment (Figure 4a). At ZT15 (Zeitgeber time; the time of day under LD conditions), food was removed or a mock food removal implemented to control for effects of disturbing the animals during the food removal [36] (Figure 4a). At the beginning of day 2, rather than the lights coming on, the mice remained in DD. Mice both with and without food were then harvested at CT5 and CT15 on day 2, and their livers extracted for analysis (Figure 4a). These times were chosen as they defined a selection of our validated exons with robust differences in alternative splicing and transcript-level expression (the exons identified within *Npas2*, *Hlf*, *Usp2*, and *Pcsk4*). Thus, we examined the response to fasting of circadian alternative splicing and transcript-level expression of these four exons. All four exons recapitulated the circadian pattern observed previously in the presence of food (Figure 4b). Under fasting conditions, however, three of the four exons showed a significant decrease in the difference between the two time points (*Npas2*, *Hlf *and *Pcsk4*; significant main effect of time and significant interaction effect between time and feeding by two-way ANOVA (*P *≤ 0.05)). Moreover, whereas a lesser temporal difference persisted with *Npas2 *and *Hlf*, no significant difference was apparent for *Pcsk4 *under fasting conditions (*P *≥ 0.05, Bonferroni's *post hoc *test). The circadian difference in *Usp2 *alternative splicing, however, was resistant to the fasting conditions (Figure 4b, inner right panel; significant main effect of time by two-way ANOVA (*P *≤ 0.05), but no significant interaction effect between time and feeding (*P *≥ 0.05)). Thus, the absence of food modulates the circadian regulation of alternative splicing in an exon-dependent manner.

The transcript-level changes observed in the presence of food faithfully recapitulated those observed previously (Figure 4c). Strikingly, however, changes in response to fasting at the transcript level did not always mirror those in alternative splicing (Figure 4c). Similar to its alternative splicing, *Usp2 *transcript-level expression was also resistant to fasting conditions, showing no significant change in the circadian difference in expression (significant main effect of time by two-way ANOVA (*P *≤ 0.05), but no significant interaction effect between time and feeding (*P *≥ 0.05)). Furthermore, *Hlf *transcript-level expression behaved similarly to its alternative splicing, being reduced by the fasting conditions. However, while some temporal difference in alternative splicing remained, the difference in transcript-level expression became insignificant (*P *≥ 0.05, Bonferroni's *post hoc *test). Given the concurrent change in both alternative splicing and transcript-level expression in response to the fasting conditions for this gene, this case would be consistent with some form of coupling between transcription and splicing. However, while *Npas2 *and *Pcsk4 *both showed a significant change in alternative splicing, neither showed a significant change in transcript-level expression (significant main effect of time by two-way ANOVA (*P *≤ 0.05), but no significant interaction effect between time and feeding (*P *≥ 0.05)), despite an apparent downward trend in *Pcsk4*. This would suggest that fasting decouples any relationship between transcription and splicing that may exist for these exons. Taken together, the molecular response to fasting in liver appears to be complex: not only do fasting conditions modulate circadian alternative splicing in an exon-dependent manner, but they also modulate the temporal relationship between circadian alternative splicing and circadian mRNA abundance in a gene-dependent manner.

### Control of circadian exons by the local liver clock

Besides the influence of direct entraining stimuli such as feeding, rhythmic gene expression in a peripheral organ can be controlled by both the local circadian clock and systemic signals emanating from the master clock of the SCN [37]. To discern whether our validated exons were being influenced by such systemic signals, we examined their expression in mice lacking the vasoactive intestinal peptide receptor 2 (*Vipr2^-/-^*). The neuropeptide Vip and its receptor Vipr2 are essential for the proper functioning of the SCN network; the SCN of mice lacking *Vipr2 *display disorganized, low amplitude rhythms in both gene expression and electrical activity [38]. Behaviorally, *Vipr2^-/- ^*mice appear rhythmic under LD, but when released into DD they show a phase-advance in locomotor activity and may become arrhythmic [39]. Remarkably, those mice that exhibit this locomotor phase-advance also display phase-advanced and fully rhythmic clock gene expression in liver. Thus, if alternative splicing and transcript-level expression of our validated exons were phase-advanced, we could consider that they are under the control of the local liver clock.

**Figure 5:**
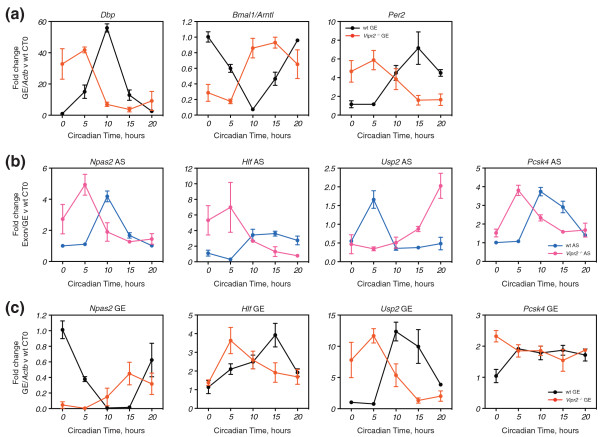
**Regulation of circadian exons in the *Vipr2*^-/- ^mouse liver**. **(a) **Expression of *Dbp*, *Per2 *and *Arntl *(*Bmal1*) in wild-type (wt) and *Vipr2^-/- ^*mouse liver over circadian time as measured by QPCR (GE, gene expression). **(b) **Alternative splicing (AS) for four selected circadian exons. **(c) **Transcript-level expression for the same exons (GE, gene expression). Expression is expressed as fold-change relative to the wild-type sample at CT0. At each time point, *n *= 3 and error bars represent standard error of the mean. In all cases, two-way ANOVA revealed a significant (*P *≤ 0.05) interaction effect between time and genotype. Subsequent one-way ANOVA of individual time-courses revealed a significant effect of time in all cases, reflecting their phase-advance, apart from *Pcsk4 *gene expression in the *Vipr2^-/- ^*mutant, reflecting its apparent arrhythmia.

Phase-advanced male *Vipr2^-/- ^*mice and wild-type controls in the second day of DD were harvested every 5 hours for one day (CT0, CT5, CT10, CT15 and CT20) and their livers extracted. Expression of core clock genes was phase-advanced by 5 to 10 hours (Figure 5a), as described previously [39]. Thus, we examined the alternative splicing and transcript-level expression of a selection of our validated exons that had previously shown robust changes around these time points. For *Npas2*, *Hlf *and *Usp2*, both alternative splicing (Figure 5b) and transcript-level expression (Figure 5c) were phase-advanced by 5 to 10 hours, indicating that both mRNA levels and alternative splicing are under the control of the local liver clock. The phase-relationships between alternative splicing and transcript-level expression observed previously were maintained in the phase-advanced context. However, this was not the case for *Pcsk4*; while alternative splicing was phase-advanced by 5 hours, transcript-level expression, which was circadian in wild-type mice, now appeared to be arrhythmic (Figure 5c, far right; *P *> 0.05, one-way ANOVA for effect of time). Although this may be an artifact of our sampling frequency, it suggests that *Pcsk4 *alternative splicing, but not transcript-level expression, is dependent on the local liver clock. Thus, we conclude that all of our tested exons are under the control of the local liver clock, but that *Pcsk4 *appears to illustrate a class of gene that integrates local and systemic circadian cues at different levels of gene expression.

### Circadian splicing factors are differentially regulated in the liver of the *Vipr2*^-/- ^mouse

**Figure 6:**
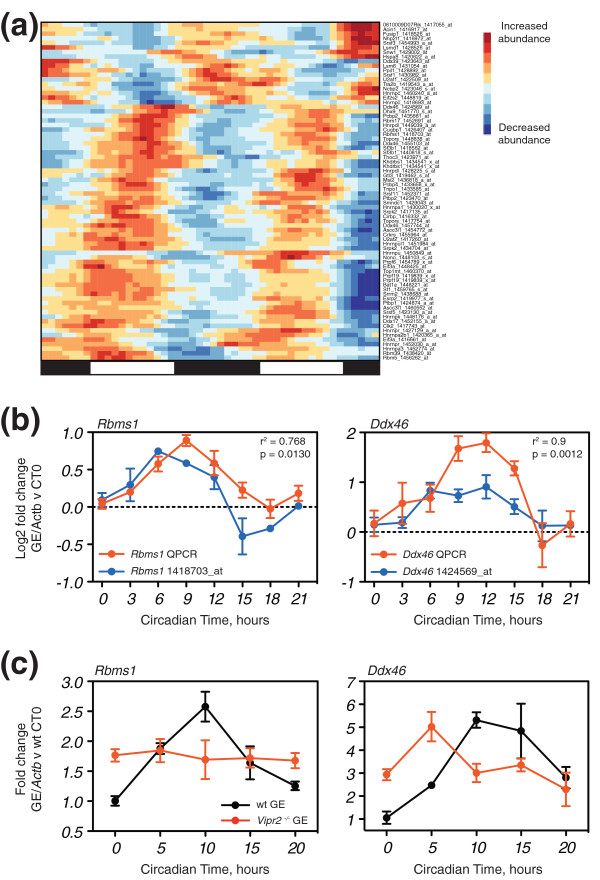
**Regulation of splicing factor expression by the circadian clock**. **(a) **Heat-map illustrating the circadian expression profiles of the probesets corresponding to the 62 splicing factors (SFs)/RNA binding proteins (RBPs) from Grosso *et al. *[40] that were defined as significantly rhythmic in Hughes *et al. *[41]. For each probeset, the RMA abundances were smoothed with a five point moving average, then rescaled to a z-score relative to the time-course. For each gene, highly similar probesets were removed. Similar circadian expression profiles were clustered on the heat-map using hierarchical clustering. **(b) **Plots of QPCR and microarray data through time for two exemplar rhythmic SFs, *Ddx46 *and *Rbms1 *(see Figure S5 in Additional file 1 for the others). QPCR data: *n *= 6 and standard error of the mean (SEM). Microarray data: equivalent circadian times in the two days were averaged and expressed relative to CT0 on the first day. Only time points with equivalents in the QPCR dataset were plotted. Error bars represent SEM. Inset: r^2 ^and *P*-value associated with Pearson correlation between QPCR and microarray data. **(c) **Expression patterns of *Ddx46 *(left, example of phase-advanced group) and *Rbms1 *(right, example of arrhythmic group) in the *Vipr2^-/- ^*liver. In each case, *n *= 3 and SEM. GE, gene expression; wt, wild type.

As discussed, exon inclusion can be regulated by a variety of mechanisms, and amongst these is the expression and activity of the families of auxiliary splicing factors. Although the expression and activity of splicing factors can be regulated by a variety of mechanisms, we hypothesized that some splicing factors undergo circadian changes in mRNA expression. To examine whether this is the case, we examined the expression of known and putative splicing factors, as defined by Grosso *et al. *[40], in a high temporal resolution microarray study of circadian mouse liver [41]. Of the 227 splicing factors, 62 had corresponding probesets judged to be rhythmic. Their circadian expression patterns showed a variety of phases, but with clear clusters of factors that peaked in expression during the subjective day and the subjective night (Figure 6a). Furthermore, these 62 were significantly more likely (*P *= 0.029, Fisher's exact test) than a random selection of 62 non-rhythmic splicing factors to contain one or more of the consensus motifs corresponding to circadian transcription factor bindings sites (E-box, E'-box, D-box and Rev response element (RRE)) in their proximal promoter region (Additional file 6), further supporting their classification as circadian.

To identify robustly circadian splicing factors, we sorted the microarray expression profiles based on their signal-to-noise ratio and the reproducibility observed between the two days. We examined the expression of the 33 best candidates by QPCR. We then compared our QPCR data to the microarray data to see whether the two temporal patterns correlated significantly (*P *≤ 0.05, Pearson's *r*); this yielded a shortlist of 12 factors (Figure 6b; Additional file 7; Figure S5 in Additional file 1). To expand our analysis, we selected three additional genes (*Pcbp2*, *Srsf3 *and *Srsf5*), bringing our shortlist to 15. These additional genes possessed significant circadian expression by QPCR in our samples (*P *≤ 0.05, one-way ANOVA for effect of time), but did not correlate significantly with the data from Hughes *et al. *[41] (Figure S5 in Additional file 1j, l). Our short-list contained a variety of genes. They include well characterized regulators of alternative splicing (*Srsf3*, *Srsf5*, *Tra2b *and *Khdrbs1 *(*Sam68*)), a component of U2 snRNP (*Sf3b1*), two RNA helicases (*Ddx46 *(also a component of U2 snRNP) and *Dhx9*), three hnRNP proteins better known for their roles regulating RNA stability and translation (*Hnrnpdl*, *Cirbp *(hnRNP-A18) and *Pcbp2 *(hnRNP E2)), and six other proteins with less well characterized roles in RNA processing (*Gtl3*, *Rbms1*, *Thoc3*, *Pcbp4 *and *Topors*).

To test if the 15 robustly circadian splicing factors were under the control of the local liver clock, we examined by QPCR whether their expression was phase-advanced in the *Vipr2^-/- ^*liver (Figure 6c; Figure S6 in Additional file 1). Thirteen of the genes showed a significant main effect of genotype and/or a significant interaction effect between genotype and time by two-way ANOVA (*P *≤ 0.05), indicating the *Vipr2^-/- ^*mutation was having an effect on the splicing factor expression. Of these 13 genes, 12 showed a significant effect of time in the wild-type time-course (*P *≤ 0.05, one-way ANOVA), confirming their normal circadian expression. Strikingly, we observed a variety of responses to the *Vipr2^-/- ^*mutation. Three of 13 genes showed a significant effect of time (*P *≤ 0.05, one-way ANOVA) in the *Vipr2^-/- ^*time-course and were clearly phase-advanced (*Ddx46*, *Pcbp2 *and *Topors*; Figure 6c; Figure S6 in Additional file 1). A further four genes showed temporal patterns very suggestive of phase-advancement, but failed to achieve statistical significance (*Srsf3*, *Cirbp*, *Sf3b1 *and *Gtl3*; Figure S6 in Additional file 1). Amongst these, *Cirbp *is of particular interest. Circadian Cirbp expression was found to be rendered arrhythmic in *Clock *mutant liver [42] (Figure S7 in Additional file 1), indicating that it is under direct clock control. However, it was also found to remain rhythmic upon specific inactivation of the liver clock [37], indicating peripheral circadian cues are sufficient to drive its rhythmicity. The remaining six genes showed no significant effect of time and appeared to be arrhythmic in the *Vipr2^-/- ^*time-course, exemplified by *Rbms1 *(Figure 6c; Figure S6 in Additional file 1). Of these genes, *Dhx9 *was also found to be arrhythmic in Clock mutant liver [42] (Figure S7 in Additional file 1). Arrhythmic expression has not been observed previously in the *Vipr2^-/- ^*mutant. Taken together, this indicates that some of the examined circadian splicing factors are under the control of the local liver clock, while others are likely rhythmic in response to systemic rhythmic cues absent in the *Vipr2^-/- ^*mutant. Those that phase-advance would be candidate regulators of the exons examined in Figure 5. As most RNA binding proteins are multi-functional [43], this indicates that the liver contains at least two circadian post-transcriptional programs, differentially dependent on the functional integrity of the SCN network.

### Candidate regulators of circadian alternative splicing

In order to identify the candidate regulators of circadian alternative splicing, we examined the correlation between the average temporal expression patterns of the robustly circadian splicing factors and the validated circadian exons (Figure S8 in Additional file 1). Several interesting associations were revealed. The strongest positive correlations were found between the *Srsf3 *splicing factor and the circadian exons within *Pcsk4 *and *Npas2 *(0.904 (*P *= 0.002) and 0.841 (*P *= 0.0089), respectively). Moreover, both these exons and *Srsf3 *expression were phase-advanced in the *Vipr2^-/- ^*mutant (compare Figure 5b and Figure S6e in Additional file 1). Strong negative correlation might indicate a repressive regulatory role for a particular factor on a particular exon. The strongest negative correlations were observed between *Srsf3 *expression and *Cabc1 *and *Clock *exon-specific expression (-0.929 (*P *= 0.0008) and -0.866 (*P *= 0.0054), respectively). These associations, both positive and negative, provide leads for further study of the molecular mechanisms controlling circadian alternative splicing.

Of particular interest are *Khdrbs1 *(*Sam68*) and *Tra2b*, which have a well-characterized role in regulating alternative splicing. To gain further insight into whether these factors may be driving circadian alternative splicing, we examined circadian splicing of a selection of their proven murine target exons. While none of the Tra2b target exons we examined were rhythmic, three of the Sam68 target exons identified by Chawla *et al. *[44] had circadian splicing patterns (Figure S9 in Additional file 1). All three showed highly significant circadian patterns by ANOVA. *Ktn1 *exon2 shows a similar circadian splicing pattern to *Rev3l *exon2, with higher exon inclusion during the late subjective day, while Opa1 exon2 shows an anti-phasic pattern. Of the three exons, only *Ktn1 *exon2 is changing with a pattern that is consistent with both the circadian expression pattern of Sam68 and the effect on the exon of Sam68 knockdown in Chawla *et al. *[44]. This may be an effect of the different biological contexts we are examining (circadian liver tissue compared to neuroblastoma cell line). In conclusion, taken together, our correlation analysis and examination of known target exons provides a list of interesting candidates for further study of the molecular mechanisms regulating circadian exons.

## Discussion

Our results demonstrate that mammalian alternative splicing is regulated by both the circadian clock and feeding. Regulation by such oscillating physiological processes adds a novel temporal dimension to the regulation of alternative splicing, distinct from that involved in both acute and chronic responses to the environment. Circadian regulation of alternative splicing occurred in a tissue-dependent manner and, similarly to circadian mRNA abundance, we anticipate that the circadian regulation of alternative splicing will be important to tissue identity and function. Moreover, the circadian clock frequently modulates physiological systems so that they respond differently to acute stimuli at different times of the day. For example, mice display circadian variation in the ability to consolidate memories in response to hippocampal-dependent fear conditioning, responding less well when conditioned during the subjective night [45]. Furthermore, the susceptibility of mice to *Escherichia coli *endotoxin-induced mortality shows a marked circadian rhythm [46]; this is reflected by circadian variation in the amount of pro-inflammatory cytokines (TNF-α and IL-6) produced by splenic macrophages in response to challenge with bacterial endotoxin (lipopolysaccharide) [47]. It will be interesting to see if this is the case for the splicing changes evoked by various acute stimuli.

We found that most of our validated exons were regulated at the levels of both alternative splicing and transcript expression. This is in contrast to what has been described for tissue-dependent alternative splicing, wherein tissue-specific exons are rarely present in genes subject to transcript-level regulation in the same tissue [7,48]. The rate of transcription can affect alternative splicing choices [49]. Therefore, the temporal co-ordination between transcription and splicing observed for our exons begs the question as to whether the two processes are coupled here. While alternative splicing and transcript-level expression changed in concert for the majority of exons tested in the *Vipr2^-/- ^*mutant, we find it unlikely that circadian transcription and splicing are coupled in *Npas2*, *Pcsk4 *and *Hlf*. This is based primarily on the result of the fasting experiment, wherein circadian variation in alternative splicing and transcript-level expression in the same gene showed differing responses to fasting conditions. However, we speculate that coupling is likely for genes such as *Usp2*, where the phase relationship between transcription and alternative splicing is preserved between tissues. Herein, both circadian alternative splicing and transcript-level expression changed in concert in the *Vipr2^-/- ^*mutant, and, moreover, neither was affected by the fasting conditions.

Another potential explanation for the co-variation of circadian alternative splicing and transcript levels is that alternative transcripts have different stability, as is the case for the *Arabidopsis *RNA binding protein (RBP) gene *Atgrp7 *[50]. One mechanism by which this can occur is via frameshifts introduced by an exon of length not divisible by three. The frameshift may lead to the creation of a premature termination codon, which targets the transcript for destruction by nonsense-mediated mRNA decay (NMD) [51]. Of our nine validated internal cassette exons, six have a length not divisible by three, indicating that their skipping would change the reading frame of the open reading frame (Additional file 2). Of these six, the circadian exons within *Loxl4 *and *Clock *are the penultimate exon, and therefore any premature termination codon created by the skipping of the exon would not be recognized as such. Of the remaining four, skipping of the circadian exon within *Pcsk4 *creates an entirely new carboxy-terminal sequence; in *Usp2 *it creates a distinct amino terminus, whereas the remaining two cassette exons (in *Npas2 *and *Fbxo21*) have the potential to create NMD-sensitive transcripts. In the case of these two exons, however, we did not find a strong negative correlation between alternative splicing and transcript-level expression (Figure S3 in Additional file 1). Taken together, this indicates that, for our validated exons, circadian alternative splicing resulting in NMD does not play a major role in the regulation of circadian transcript abundance. However, it may be the case that both circadian alternative splicing resulting in NMD and circadian transcription are necessary for high-amplitude oscillation in transcript levels, as has shown to be the case for the *Arabidopsis *RBP Atgrp7 [50].

Circadian regulation of alternative splicing was shown to be modulated by fasting conditions in an exon-dependent fashion. To our knowledge this is the first demonstration that fasting causes changes in alternative splicing. Previous studies have shown that a similar fasting regime affects the efficiency of splicing of particular constitutive introns of glucose-6-phosphate dehydrogenase (*G6pdx*) [52,53], and also that long term changes in larval nutrition can affect *troponin-t *alternative splicing in the flight muscles of the moth *Spodoptera frugiperda *[11]. Besides their effect on circadian alternative splicing, fasting conditions also appear to modulate the temporal relationship between circadian alternative splicing and circadian transcript abundance in a gene-dependent manner. With our limited temporal resolution, we are unable to say whether these changes represent decreases in the amplitude of the circadian change or phase shifts. A previous study examining the effect of fasting on circadian transcript-level expression in the liver observed that only a minority of rhythmic transcripts remained so after fasting, and those that remained rhythmic showed a reduction in amplitude [35]. This is consistent with some of our results, although we clearly see genes whose expression appears resistant to fasting (*Npas2 *and *Usp2 *particularly; Figure 4b). Moreover, we also examined the expression of a panel of core clock genes under our fasting conditions by QPCR (Figure S10 in Additional file 1). We observed an apparent decrease in amplitude for *Cry2*, while both *Cry1 *and *Per2 *appeared unaffected statistically, and the amplitude of the circadian difference for *Bmal1 *and *Dbp *appeared to have increased. One possible explanation is that the fasting paradigm employed by Vollmers *et al. *[35] is very different to that used here, with the samples for subjective day and subjective night being taken from separate animal experiments to preserve an equivalent amount of fasting in each case. More similar was the paradigm used by Ishida and colleagues [54], though fasting was performed under LD conditions. In their study, on the first day of fasting, the *Per2 *expression pattern was essentially unchanged, though this was also the case for *Dbp*. It may be that the LD conditions are sufficient to cause this difference.

Using the *Vipr2^-/- ^*mouse, we observed that all our tested exons were phase-advanced in the liver under DD (Figure 5b). This was also the case for the corresponding transcript-level expression (with the notable exception of *Pcsk4*; Figure 5c), and a panel of core clock genes (Figure 5a). While the simplest explanation for these observations is that the phase-advancing exons and transcript-level expression are under control of the advanced local liver clock, it is also formally possible that the phase-advance represents a direct response to specific systemic circadian signals absent in the *Vipr2^-/- ^*mouse, i.e. that phase-advance in the absence of a functional SCN network. The arrhythmic response of *Pcsk4 *transcript-level expression was generalized when we observed that some rhythmic splicing factors phase-advance, while others become apparently arrhythmic in the *Vipr2^-/- ^*mutant (Figure 6c; Figure S6 in Additional file 1). This suggests to us that there are two circadian post-transcriptional programs in liver; one controlled by the local clock and the other independent of it, perhaps controlled by systemic circadian cues absent in the *Vipr2^-/- ^*mutant. Examining a greater number of exons in an extended time-course would likely reveal exons that were arrhythmic in the *Vipr2^-/- ^*mutant. Indeed, in the future, more sensitive transcriptomic approaches (such as RNA-seq) and the use of mice lacking core clock genes (such as the *Clock *and *Bmal1 *knockouts) will likely uncover the full diversity of alternative splicing events regulated by the clock. Combining these observations with those from the fasting experiment indicates that single genes can integrate circadian information from multiple sources and responses to the environment (that is, the absence of food) at different levels of regulatory control. Indeed, the contrast between the uniform changes in alternative splicing observed in the *Vipr2^-/- ^*mutant with the variety of responses to the fasting conditions suggests that the effect of fasting is being mediated through a pathway other than the liver clock.

Our results suggest that a comparatively small proportion of genes contain circadian exons. This view is supported by a recent RNA-seq study examining the diurnal and circadian transcriptome of the *Drosophila *brain [55]. However, neither study had a high sampling frequency in their circadian time-course. Based on the number of genes with circadian exons identified by the exon-array in liver, and the relationship between sampling frequency and number of circadian transcripts described by Hughes *et al. *[41], we speculatively estimate that approximately 20% of mouse genes contain a circadian exon (see Supplementary Experimental Procedures in Additional file 1 for details of how this estimate was derived). Furthermore, given our demonstration that circadian alternative splicing is frequently tissue-dependent, the true figure may be somewhat higher than this. While this remains comparatively infrequent compared to tissue-dependent alternative splicing, the functional impact of circadian alternative splicing, and its contribution to circadian phenotypes, may still be large. Alternative splicing can result in drastic differences in the molecular function and interactions of the resultant isoforms [56]. Indeed, the ratio between isoforms is often in itself an important factor in a gene's function [56]. Circadian alternative splicing could specify time-of-day-dependent protein functions or interaction partners, whereas circadian variation in isoform frequency could mediate a temporal bias of a 'decision-making' pathway (for example, apoptosis) towards a particular outcome. Of our validated circadian cassette exons, three are part of annotated protein domains. *Loxl4 *and *Pcsk4 *both encode enzymes: Loxl4 is a lysyl oxidase; Pcsk4 is a proprotein convertase, belonging to a family of calcium-dependent serine proteases that proteolytically process precursor proteins (typically hormones and growth factors). In both genes, the circadian exon is one of a group that encodes a domain essential for the activity of the enzyme (the P domain of Pcsk4, and the lysyl oxidase domain of Loxl4 [57,58]). Therefore, we might expect that alternative splicing of this exon would have a significant effect on the activity of the enzyme, perhaps reducing its activity, or changing its substrate specificity. We also identified *Npas2 *and *Clock *as containing circadian exons (Figure S3a in Additional file 1). Npas2 is a bHLH (basic helix-loop-helix) transcription factor and, interestingly, a functional analog of the core circadian gene *Clock*. The circadian exon is within the protein's PAS A domain, the integrity of which has been shown to regulate the protein's interaction with Arntl (Bmal1), and its transcriptional activity in NIH 3T3 cells [59]. Across the phyla, many proteins involved in the circadian clock contain PAS domains [59]. In mammals they have also been shown to mediate homo- and heterodimerization of the three mammalian Period proteins. Indeed, mice carrying a point mutation of the PAS B domain of Per2 display a short period followed by arrhythmicity under DD, while mice lacking the domain entirely are prone to cancer [60]. Thus, we find it likely that an isoform of Npas2 lacking the PAS A domain would have altered transcriptional activity. The periodic expression of this isoform could be a mechanism to diversify the circadian transcriptional program specified by Npas2. The same may also be true of Clock. The circadian exon is within a glutamine-rich low-complexity region towards the carboxyl terminus of the protein. Glutamine-rich regions often mediate protein-protein interactions, and the same region in the *Drosophila *Clock ortholog (CLK) has been implicated in mediating transcriptional trans-activation by the CLK-CYC heterodimer [61,62]. Lastly, *Pnp1*, a gene involved in purine nucleoside metabolism, provides another interesting example. Our data indicate that *Pnp1 *is undergoing circadian alternative polyadenylation between a short and long form of the 3' UTR (Figure S3c in Additional file 1). 3' UTRs can contain many sequence features that regulate mRNA stability, localization and the tendency to be translated, particularly microRNA (miRNA) binding sites and A/U rich elements (AREs). Indeed, it has been shown that cellular proliferation is associated with a general shortening of 3' UTR lengths through alternative polyadenylation, and that this reduces the amount of miRNA-mediated regulation [63]. It is attractive to speculate that a similar situation is occurring for *Pnp1 *mRNA on a circadian timescale. While we were unable to identify any miRNA binding sites within the long portion of the 3' UTR, the region is poorly conserved outside of rodents, which would confound many of the better algorithms for miRNA binding site prediction. Moreover, the anti-phasic relationship between a QPCR primer specific for the long form of the 3' UTR and that measuring whole transcript levels is very suggestive of a form of UTR-mediated regulation involving mRNA stability (Figure S3c in Additional file 1). This is in contrast to the circadian alternative polyadenylation occurring in *Decr1*, where the transcript-level expression shows no circadian pattern. Here the alternative portion of the UTR may contain an element that regulates translation without affecting RNA stability. Similarly to *Pnp1*, the alternative portion of the UTR is poorly conserved outside of rodents.

Though alternative splicing can be regulated in many ways, the identification of 15 robustly circadian splicing factors/RBPs suggests that circadian splicing factor abundance may direct circadian alternative splicing. This idea is lent further weight by our finding that a number of previously identified target exons of the circadian splicing factor Sam68 are rhythmic in liver. Many of the robustly circadian splicing factors/RBPs do not have a well-characterized role in regulating alternative splicing. However, we found that the expression of many of these factors correlates strongly with our validated circadian exons. Thus, it will be interesting to examine their physiological binding targets, using techniques such as iCLIP [64], and how their rhythmic abundance might regulate alternative splicing on a circadian timescale.

Sanchez *et al. *[22] recently demonstrated that the *Arabidopsis *protein methylase PRMT5, and its *Drosophila *ortholog dart5, regulates both circadian alternative splicing and period length. This indicates the existence of a feedback loop wherein the clock regulates alternative splicing and, in turn, is regulated by alternative splicing. Our identification of circadian exons within *Clock*, *Npas2 *and *Nr1d1 *suggests the same may also be true in mammals. This strikes us as a further example of a recurrent motif in circadian biology, wherein circadian outputs feedback to regulate the clock itself. This is exemplified by the clock's control of metabolism. For example, circadian production of the cellular metabolite nicotinamide adenine dinucleotide (NAD+) feeds back onto the clock through promoting the action of the deacetylase Sirt1, which appears to work both in opposition to Clock and to destabilize Bmal1 and Per2 [13]. This feedback is thought to buffer the clock to changes in the metabolic state of the cell. Interestingly, mutation of PRMT5/dart5, and the resulting changes in alternative splicing, changes the period of behavioral rhythms [22]. This suggests that the feedback provided by alternative splicing may be providing some other function than buffering. To further examine the role of alternative splicing in the mammalian clock, it will be of great interest to examine the circadian phenotypes of mice conditionally lacking the regulators of alternative splicing.

## Conclusions

Our results demonstrate that the circadian clock regulates alternative splicing in the mouse. This uncovers a novel temporal dimension to the regulation of alternative splicing and indicates circadian biology is a system within which to study the links between physiology, behavior and alternative splicing (or, indeed, other post-transcriptional regulatory mechanisms). Our data indicate that circadian alternative splicing is tissue-dependent and can occur concurrently with circadian transcript abundance. Moreover, we show that circadian alternative splicing, and its relationship with circadian transcript abundance, can be modulated by fasting. Taken together with our results from *Vipr2^-/- ^*mice, this indicates that circadian alternative splicing is regulated by the interaction between distinct physiological cues, and illustrates the capability of single genes to integrate circadian signals at different levels of regulation. Finally, our discovery of robustly circadian splicing factors, and that a number of their previously characterized target exons are circadian, provides candidates for further study into the molecular mechanisms regulating circadian exons and other post-transcriptional processes.

## Materials and methods

### Animal experiments

Studies conformed to the Animals (Scientific Procedures) Act (1986). Adult male wild-type (C57BL/6) and *Vipr2^-/- ^*(C57BL/6 background) mice were housed under 12 h:12 h light:dim red light (LD) with *ad libitum *food and water. For circadian locomotor activity recording, mice were caged within light-tight ventilated cabinets and transferred from LD to continuous dim red light (DD). Locomotor behavior was analyzed using ClockLab software (ActiMetrics, Wilmette, IL, USA). The exon-array and validation experiments were performed simultaneously. Herein mice were caged in groups of four to five during behavioral analysis due to the large number of mice involved [23,65]. In the experiment comparing wild-types to *Vipr2^-/- ^*mutants (Figures 5 and 6), mice were individually caged during behavioral analysis. To obtain circadian timed organ samples, mice at the appropriate time were harvested under DD and extracted organs were rapidly frozen on dry ice, then stored at -80°C. Total RNA was extracted from frozen tissue by homogenization in Tri-Reagent using a rotor-stator homogenizer, followed by purification using the Ribopure kit (Life Technologies (Grand Island, NY, USA). RNA was then treated with the TURBO DNA-*free *kit (Ambion).

### Exon-array analysis

#### Target preparation and analysis

The purity and integrity of total RNA samples were confirmed using scanning spectrophotometry (Nanodrop, LabTech) and capillary electrophoresis (RNA 6000 Nano chip on 2100 Bioanalyser, Agilent Technologies, Santa Clara, CA, USA). For each sample, 2 μg total RNA was depleted of ribosomal RNA (Ribominus, Life Technologies, Grand Island, NY, USA) and amplified using a random priming technique (WT Expression kit, Affymetrix, Santa Clara, CA, USA) following the manufacturer's instructions. The fragmented biotin-labeled libraries produced were hybridized overnight to mouse Exon ST 1.0 arrays (Affymetrix) before staining and scanning on a GCS 3000 microarray scanner (Affymetrix). The raw exon-array data have been deposited into the arrayExpress database under the accession E-MEXP-3644

#### Data pre-processing and FIRMA analysis

Exon-array data were pre-processed and subjected to FIRMA analysis using the *aroma.affymetrix *package in R, as described in the Human exon-array analysis vignette [27,66,67]. A custom chip description file covering the exon-array core probesets was used (MoEx-1_0-st-v1, coreR1, A20080718, MR.cdf). In order to identify exons regulated by the circadian clock, we sought to identify exon-probesets undergoing circadian variation in their FIRMA score. To this end, we identified exon-probesets whose FIRMA score was changing significantly (FDR-adjusted *P *< 0.05) as a function of time using a one-way ANOVA implemented in *limma *[68]. To exclude exon-probesets undergoing non-circadian changes, we used *limma *to identify exon-probesets whose FIRMA scores differed significantly (FDR-adjusted *P *< 0.05) in the comparison [CT0 and CT12] versus [CT6 and CT18], then removed these from the list of exon-probesets changing as a function of time. The remaining exon-probesets constituted our predicted circadian exons (Additional file 2).

### Analysis of mRNA expression by RT-PCR and QPCR

#### Reverse transcription

DNase-treated total RNA was reverse transcribed with Superscript 3 (Life Technologies) using a combination of random hexamers and anchored oligo-dT primer (Life Technologies).

#### Quantitative PCR

QPCR was performed on a Rotor-Gene 6000 (Qiagen, Hilden, Germany) using SYBR Green JumpStart Taq ReadyMix (Sigma, St. Louis, MO, USA). QPCR data were analyzed using the comparative concentration module of the Rotor-Gene software, which is based on [69].

#### Transcript-level expression

Signal for the gene of interest was normalized to signal for *Actb*, then fold change was calculated relative to CT0. For alternative splicing, an exon-specific primer pair was normalized to that measuring transcript-level expression. For primer sequences see Table S7 (Additional file 8). For each primer pair, the formation of a single product was confirmed by melt curve analysis [70].

#### RT-PCR

PCR was performed with primers flanking the predicted alternative exon, using BIOTaq polymerase (Bioline, London, UK) or Phusion Flash High-Fidelity PCR Master Mix (ThermoFisher, Waltham, MA, USA) according to the manufacturers' instructions. The QIAxcel capillary gel electrophoresis system (Qiagen) was used to visualize RT-PCR products. Percentage exon inclusion was determined as the size-corrected normalized area of the exon-included band divided by the sum of signals for exon-included and exon-skipped bands. All primers used in this study are detailed in Additional file 8.

### Bioinformatics

#### Prediction of clock transcription factor binding sites associated with (ar)rhythmic splicing factors

A selection of arrhythmic splicing factors was taken from the list of known and putative splicing factors provided by Grosso *et al. *[40], using the R function *sample *(without replacement). For each splicing factor of interest, we took each of the corresponding Refseq mRNAs and extracted the sense sequence from 5 kb upstream of the transcription start site to the end of the first intron. These sequences were then filtered to remove duplicates. This was performed on mouse genome assembly mm9, using Galaxy and the UCSC table browser [71-74]. Potential clock-transcription factor binding sites were predicted based on custom-made position-specific scoring matrices (PSSMs; Additional file 1) using MotifScanner, employing the default background model and examining both strands [75]. The predicted motifs are detailed in Additional file 6. For each of the rhythmic and arrhythmic splicing factor sets, the number of genes with (and without) one or more Refseq mRNAs containing one or more instances of one or more of the clock-transcription factor motifs was calculated. The presence of an enrichment in clock-transcription factor motifs in the rhythmic splicing factors was tested with Fisher's exact test (one tail).

#### Annotation enrichment analysis

Enrichment of the genes identified by the FIRMA analysis for various functional annotation terms was performed using DAVID functional annotation tools [76] and WebGestalt [77] using default settings. In both cases the background list was specified as those genes represented on the Affymetrix mouse exon-array.

## Abbreviations

ANOVA: analysis of variance; CT: circadian time; DD: constant darkness (dim red light); FDR: false discovery rate; FIRMA: Finding Isoforms using Robust Multichip Analysis; hnRNP: heterogenous nuclear ribonucleoprotein; KEGG: Kyoto Encyclopedia of Genes and Genomes; LD: lighting schedule consisting of 12 h light followed by 12 h dim red light; miRNA: microRNA; NMD: nonsense-mediated mRNA decay; QPCR: quantitative polymerase chain reaction; RBP: RNA binding protein; RMA: Robust Multichip Analysis; SCN: suprachiastic nucleus; UTR: untranslated region.

## Competing interests

The authors declare that they have no competing interests.

## Authors' contributions

NJM, JU and MHH conceived the study. JEC, ESM and MHH performed the animal handling portions of the experiments. NJM and AV performed all other portions of the experiments. NJM analyzed the data and performed the bioinformatics analyses. NJM, JU and MHH wrote the paper. The manuscript was read and approved by all authors.

## Supplementary Material

Additional file 1 **Supplemental Data**. Supplemental Figures 1 to 10 and associated legends, supplemental experimental procedures and supplemental references. Click here for file

Additional file 2 **Table S1**. Information regarding the positive probesets identified by the FIRMA analysis, compiled using the *exonmap *and *xmapcore *bioconductor packages [1-3]. *probeset *refers to the exon-probeset of interest. Fields: *sotaCluster *refers to the self-organizing tree algorithm (sota) cluster the probeset is part of (Figure 1b); *adj.P.Val.Limma *refers to the multiple-testing corrected *P*-value associated with the one-way ANOVA for an effect of time executed in *limma *[68,81] (see Materials and methods); *ensExonID *refers to the Ensembl [82] identifier for the exon within which the probeset is located; *probeLoc *refers to the mm9 genomic co-ordinates for the probeset of interest; *exonSequence *refers to the sequence of the exon identified by *ensExonID*; *divisableBy3 *indicates whether the exon's length is a multiple of three or not; *ensGeneID *refers to the Ensembl [82] identifier for the gene within which the exon identified by *ensExonID *is located; *geneLoc *refers to the mm9 genomic co-ordinates for the gene of interest; *validation *refers to validation status of the exon of interest (nd, not determined); *description *refers to official long name of the gene of interest. Click here for file

Additional file 3 **Table S2**. Summary of the functional annotation data obtained for the positive genes from the FIRMA analysis, using DAVID [76]. Fields: *Category *refers to the source of the functional annotation term; *Term *refers to the particular enriched annotation term; *PValue *refers to the raw *P*-value associated with the enrichment of the term; *Genes *refers to the gene symbols of those genes from the list that contribute to the enrichment of this term; *Fold Enrichment *refers to the level of enrichment of this term over the background distribution; *Benjamini *refers to the *P*-value for enrichment after correction for multiple testing according to the method of Benjamini and Hochberg [83]; *ofInterest *refers to whether this term was considered pertinent to our analysis. Click here for file

Additional file 4 **Table S3**. Results of the Gene Ontology term [29] enrichment analysis performed using WebGestalt [77]. Click here for file

Additional file 5 **Table S4**. Results of the KEGG pathway [28,84,85] enrichment analysis performed using WebGestalt [77]. Click here for file

Additional file 6 **Table S5**. Output of MotifScanner from the analysis of predicted clock transcription factor binding sites in the proximal promoter region of circadian splicing factors/RBPs. *refseq *refers to the Refseq mRNA relative to which the examined sequence was extracted. *location *refers to the genomic co-ordinates of the sequence examined. Click here for file

Additional file 7 **Table S6**. Correlations between microarray data from Hughes *et al. *[41] and QPCR measurements from this study for circadian splicing factors/RBPs identified from Hughes *et al. *[41]. *Quality *refers to a manual assessment of the robustness of the circadian expression profile for that probeset, 1 being best and 3 being worst. Click here for file

Additional file 8 **Table S7**. Details of all the oligonucleotides used in this study. Click here for file
