# Supplementary material for: Regulation of alternative splicing by the circadian clock and food related cues
Source: Genome Biol. 2012 Jun 21;13(6):R54. doi: 10.1186/gb-2012-13-6-r54 (PMC3446320; doi:10.1186/gb-2012-13-6-r54)
Supplement: Additional file 1 — Supplemental Data. Supplemental Figures 1 to 10 and associated legends, supplemental experimental procedures and supplemental references. [file gb-2012-13-6-r54-S1.PDF]

Supplementary Data

Supplemental Figure Legends

Figure S1. The Exon-Array Accurately Reproduces The Circadian Expression Pattern of Core Clock Genes.

Comparison of exon array and QPCR measurements for **A**. *Dbp* and **B**. *Arntl* (*Bmal1*) in the samples used for the exon-array analysis. In both cases, n=3 and error bars represent the standard error of the mean (SEM). For the exon array data, fold-change versus CT0 was calculated from the RMA abundances for each gene.

Figure S2. Circadian expression of Per2, Dbp and Arntl (Bmal1) in validation tissue sets.

Expression of *Per2* (*top panel*), *Dbp* (*middle panel*) and *Arntl* (*Bmal1*, *bottom panel*) through circadian time in liver, kidney and lung, as measured by QPCR. In each case, n=6 and error bars represent SEM.

Figure S3. Exon array validation

Validation results for **A**. *Npas2* **B**. *Usp2* **C**. *Pnp1* **D**. *Fbxo21* **E**. *Hlf* **F**. *Ash2l* **G**. *Nr1d1* **H**. *Cabc1* **I**. *Loxl4* **J**. *Decr1* **K**. *Adrbk2* **L**. *Clock*. In each case: *Top panel*. UCSC genome browser screen-shots illustrating the structure of the Refseq mRNA and the location of the candidate. *Middle panel*. Screen-shot of the closer genomic context of the identified exon-probeset, including the location of the core set of exon-array probesets and vertebrate &

euarchontoglires conservation as measured by phastCons score (white=0, black=1) [1,2]. *Bottom left panel*. Plot of the log2 FIRMA score (black line, n=3, SEM) and alternative splicing as measured by exon-specific QPCR (red line, n=6, SEM) through circadian time. *Bottom right panel*. Plot of the alternative splicing (red line, n=6, SEM, same data as the right panel) and total gene expression as measured by QPCR (blue line, n=6, SEM) through circadian time. Additionally, in **I**, the bottom-most panel shows a representative Qiaxcel gel of the RT-PCR data in the above plot.

Figure S4. Circadian expression of validated exons is tissue-dependent.

Cross-tissue validation results for **A**. *Usp2* **B**. *Hlf* **C**. *Pnp1* **D**. *Ash2l* **E**. *Nr1d1* **F**. *Fbxo21* **G**. *Cabc1* **H**. *Npas2*. *Left panel:* alternative splicing. *Right panel:* gene-level expression through circadian time (left to right), and in three visceral tissues: liver (white, same data as Figure S1), kidney (light grey) & lung (dark grey). In each case, n=6 and error bars represent SEM. Overhanging bars & asterisks indicate results of a one-way ANOVA for an effect of circadian time (* - $p \leq 0.05$; ** - $p \leq 0.01$; *** - $p \leq 0.001$). Individual tissue time-courses were examined where a two-way ANOVA had revealed a significant effect of tissue and/or an interaction between tissue and circadian time. However, this was not the case for *Ash2l* gene expression and *Cabc1* exon specific expression. In all cases there was a significant affect of circadian time.

Figure S5. SRGs with rhythmic mRNA in liver.

A.-**I.** & **M**. Plots of QPCR and microarray data through circadian time for SRGs rhythmic by QPCR that correlated significantly with the microarray data from Hughes *et al.* (2009). **J**.-**L**. Plots for those factors that showed a significant circadian expression pattern but did not correlate significantly with the microarray data. QPCR data: n=6, SEM. Microarray data: equivalent circadian times in the two cycles were averaged and expressed relative to CT0 in the first cycle. Only time-points with equivalents in the QPCR dataset were plotted. Error bars represent SEM. For each gene, the correlation between QPCR and microarray data was examined using Pearson's *r*. The results of this analysis are tabulated in Table S6.

Figure S6. Regulation of rhythmic SRGs in the Vipr2$^{-/-}$ mouse liver.

Expression patterns of rhythmic SRGs as measured by QPCR. **A**.-**F**. Examples of SRGs that appear to be phased advanced in the *Vipr2*$^{-/-}$ mouse liver compared to wild-type. **G**.-**M**. Examples of SRGs that appear to be arrhythmic in the *Vipr2*$^{-/-}$ mouse liver compared to wild-type. In each case, n=3 and error bars represent SEM.

Figure S7. Circadian expression of *Cirbp* and *Dhx9* in *Clock* mutant liver.

Circadian expression patterns of **A**. *Cirbp* and **B**. *Dhx9* in wildtype (black symbol and line) and *Clock* mutant (pink symbol and line) liver as measured by Miller *et al.* (2007). In each case, the individual observations are plotted as points with a line connecting the mean at each timepoint.

Figure S8. Correlation between validated circadian exons and splicing factors

Heatmap illustrating the correlation (Pearson's *r*) between the circadian expression profiles (log2 fold change vs. CT0) of individual validated circadian exons (y-axis) and individual robustly circadian splicing factors/RBPs (x-axis). The strongest positive correlations were found between the *Srsf3* splicing factor and the circadian exons within *Pcsk4* & *Npas2* (0.904 & 0.841, respectively).Strong positive correlations were also identified between these exons and *Thoc3* expression (0.841 & 0.810, respectively); *Hnrnpdl* expression and the alternative polyadenylation event in *Decr1* (0.806); and expression of the two helicases, *Ddx46* (0.786 with *Npas2* exon) and *Dhx9* (0.783 with *Pcsk4* exon). The strongest negative correlations were observed between *Srsf3* expression and *Cabc1* & *Clock* exon-specific expression (-0.929 & -0.866, respectively)

Figure S9. Circadian alternative splicing of Khdrbs1 (Sam68) target exons.

Circadian alternative splicing patterns of three Sam68 target exons identified by Chawla *et al.* (2009) [3]. **A**. *Rev3l*_2 **B**. *Opa1*_2 **C**. *Ktn1*_2. Inclusion levels were measured by RT-PCR using the primers detailed in Chawla *et al.* and the validation experiment samples from this study. At each timepoint, N=6 and error bars represent SEM. Circadian alternative splicing patterns were judged to be significant by ANOVA (*Rev3l*_2: $p=0.0001$, *Opa1*_2: $p<0.0001$ *Ktn1*_2: $p=0.0166$).

Figure S10 Core clock gene expression is selectively modulated by fasting conditions

In each case, expression is expressed as fold change compared to CT5. For each combination of timepoint and feeding status, n=3 and error bars represent SEM. All genes showed a significant main effect of time by two-way ANOVA ($p \leq 0.05$). Moreover, *Bmal1* and *Dbp* also showed a significant interaction effect between time and feeding ($p \leq 0.05$). The symbols above each pair of bars indicate the results of Bonferroni's *post-hoc* test, comparing the two circadian timepoints in each feeding condition after a two-way ANOVA: ns - not significant, * - $p \leq 0.05$, ** - $p \leq 0.01$, *** - $p \leq 0.001$, **** - $p \leq 0.0001$.

Supplemental Experimental Procedures

Bioinformatics

PSSMs for clock transcription factors: For each motif, each row of the matrix represents a nucleotide position (the first row representing the most 5' position etc.) and each column a different nucleotide. Thus the number at each position reflects the probability of observing that nucleotide at that position in the motif.

D-Box

letter-probability matrix: alength= 4 w= 8

A	C	G	T
0.0011	0.0005	0.0005	0.9979
0.0011	0.0005	0.0005	0.9979
0.9979	0.0005	0.0005	0.0011
0.0011	0.4978	0.0011	0.4978
0.0011	0.0559	0.9419	0.0011
0.0011	0.0005	0.0005	0.9979
0.9979	0.0005	0.0005	0.0011
0.9979	0.0005	0.0005	0.0011

E'-Box

letter-probability matrix: alength= 4 w= 6

A	C	G	T
0.0011	0.9973	0.0005	0.0011
0.9979	0.0005	0.0005	0.0011
0.0011	0.9973	0.0005	0.0011
0.0011	0.0559	0.9419	0.0011
0.0011	0.0005	0.0005	0.9979
0.0011	0.0005	0.0005	0.9979

E-Box

letter-probability matrix: alength= 4 w= 6

A	C	G	T
0.01042	0.96875	0.01042	0.01042
0.96875	0.01042	0.01042	0.01042
0.01042	0.96875	0.01042	0.01042
0.01042	0.01042	0.96875	0.01042
0.01042	0.01042	0.01042	0.96875
0.01042	0.01042	0.96875	0.01042

RRE

letter-probability matrix: alength= 4 w= 11

A	C	G	T
0.4978	0.0011	0.0011	0.4978
0.9979	0.0005	0.0005	0.0011
0.4978	0.0011	0.0011	0.4978
0.2500	0.2500	0.2500	0.2500
0.0011	0.0005	0.0005	0.9979
0.4978	0.0011	0.4978	0.0011
0.0011	0.0559	0.9419	0.0011
0.0011	0.0559	0.9419	0.0011
0.0011	0.0005	0.0005	0.9979
0.0005	0.9979	0.0005	0.0011
0.9979	0.0005	0.0005	0.0011

An estimate of the frequency of circadian alternative splicing

The core probeset of the mouse exon array comprises 229518 probsets (roughly corresponding to exons), grouped into 23332 metaprobesets (roughly corresponding to genes). To determine the number of expressed genes detected by the array, we used Affymetrix Power Tools (APT) to calculate the dabg (detection above back-ground) p-value for each of the metaprobesets derived from the core probesets on the array. The dabg p-value reflects the probability that the intensities in a probeset could have been observed by

chance given the distribution of GC-content matched background probes on the exon-array. The p-value can be used as a substitute for standard absent/present calls when mismatch probes are not available. Metaprobesets with $p \geq 0.05$ in ≥75% of the array were excluded from the analysis. From the remaining 17831 metaprobesets, we were able to unambiguously identify 15416 Ensembl genes, 13346 of which have more than two exons, making them capable of alternative splicing (86.6%, consistent with the genome-wide figure). Using the exon-array, we identified circadian exons in 47 genes, therefore 0.4% of expressed genes with greater than two exons.

To gain a speculative estimate of the total number of genes with circadian exons in liver, we used the relationship between sampling frequency and circadian transcripts detected by Fisher's G test described in Hughes *et al.* (2008) (Figure 4C therein). While the experimental design and analysis method differ greatly, six hour sampling resolution with an FDR of 0.4 resulted in a similar number of rhythmic transcripts as genes with circadian exons. If the two circadian phenomena follow a similar exponential relationship with sampling frequency, we might expect ~2500 genes with circadian exons (18.7% of expressed genes with greater than two exons) if we measure at two-hour resolution over two days. Given our demonstration that circadian alternative splicing is frequently tissue dependent, the true proportion may be somewhat higher than this.

Supplemental References

1. Siepel A, Bejerano G, Pedersen JS, Hinrichs AS, Hou M, Rosenbloom K, Clawson H, Spieth J, Hillier LW, Richards S, Weinstock GM, Wilson RK, Gibbs RA, Kent WJ, Miller W, Haussler D: **Evolutionarily conserved elements in vertebrate, insect, worm, and yeast genomes**. *Genome Res* 2005, **15**:1034–105010.1101/gr.3715005.

2. Karolchik D, Baertsch R, Diekhans M, Furey T, Hinrichs A, Lu Y, Roskin K, Schwartz M, Sugnet C, Thomas D, Weber R, Haussler D, Kent W: **The UCSC Genome Browser Database**. *Nucleic Acids Res* 2003, **31**:51–54.

3. Chawla G, Lin C-H, Han A, Shiue L, Ares M, Black DL: **Sam68 regulates a set of alternatively spliced exons during neurogenesis**. *Molecular and Cellular Biology* 2009, **29**:201–21310.1128/MCB.01349-08.

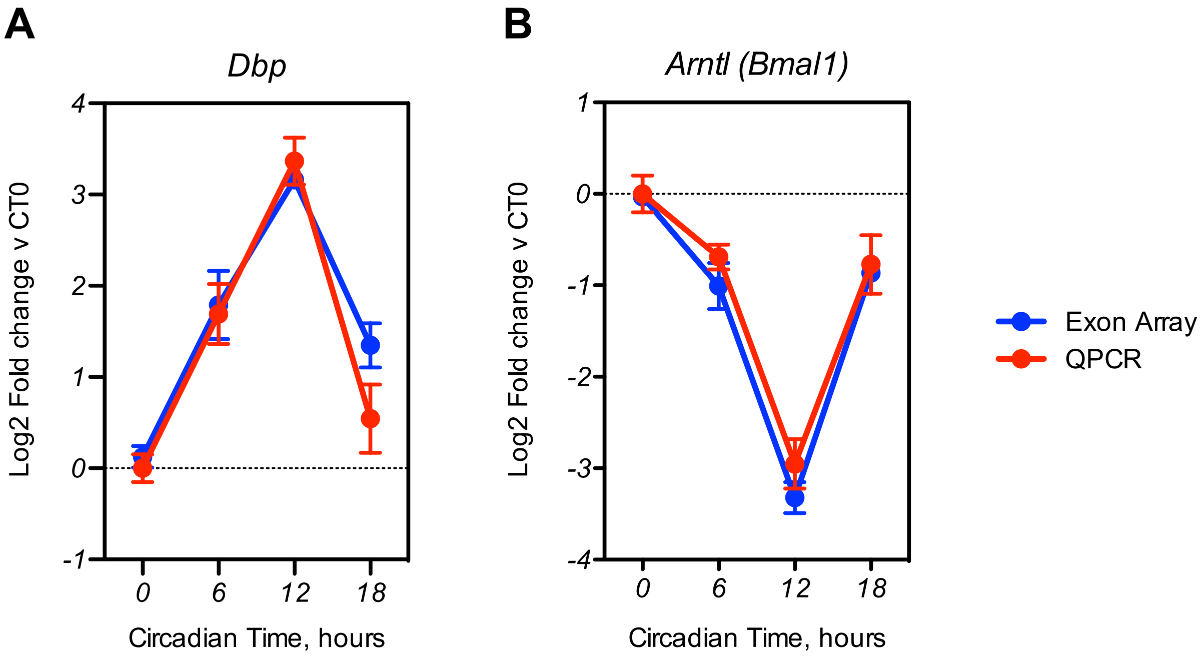
A
Dbp
Log2 Fold change v CT0
4
3
2
1
0
-1
0
6
12
18
Circadian Time, hours
B
Arntl (Bmal1)
Log2 Fold change v CT0
1
0
-1
-2
-3
-4
0
6
12
18
Circadian Time, hours
Exon Array
QPCR

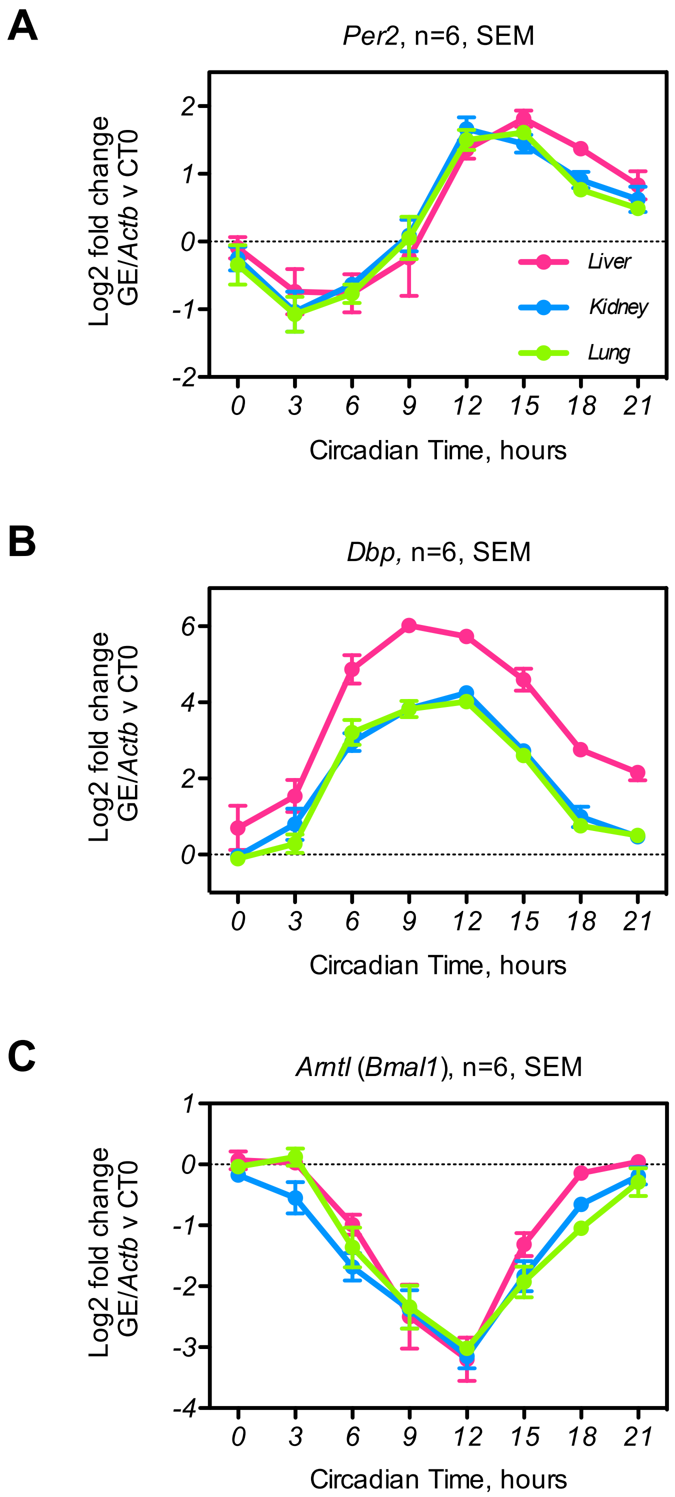
A
Per2, n=6, SEM
Log2 fold change GE/Actb v CT0
2
1
0
-1
-2
Liver
Kidney
Lung
0 3 6 9 12 15 18 21
Circadian Time, hours
B
Dbp, n=6, SEM
Log2 fold change GE/Actb v CT0
6
4
2
0
0 3 6 9 12 15 18 21
Circadian Time, hours
C
Arntl (Bmal1), n=6, SEM
Log2 fold change GE/Actb v CT0
1
0
-1
-2
-3
-4
0 3 6 9 12 15 18 21
Circadian Time, hours

mcglincy_figS3i

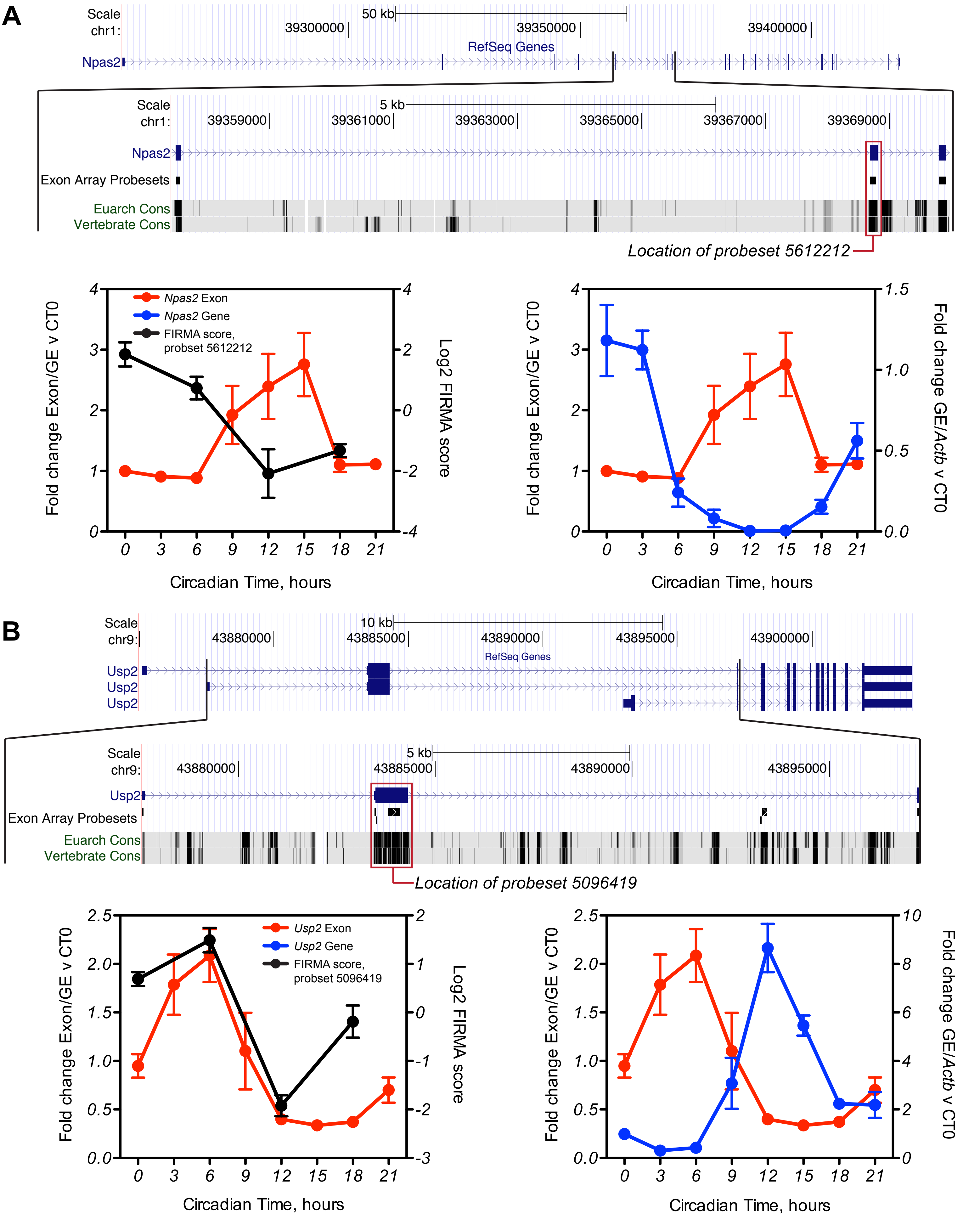

mcglincy_figS3ii

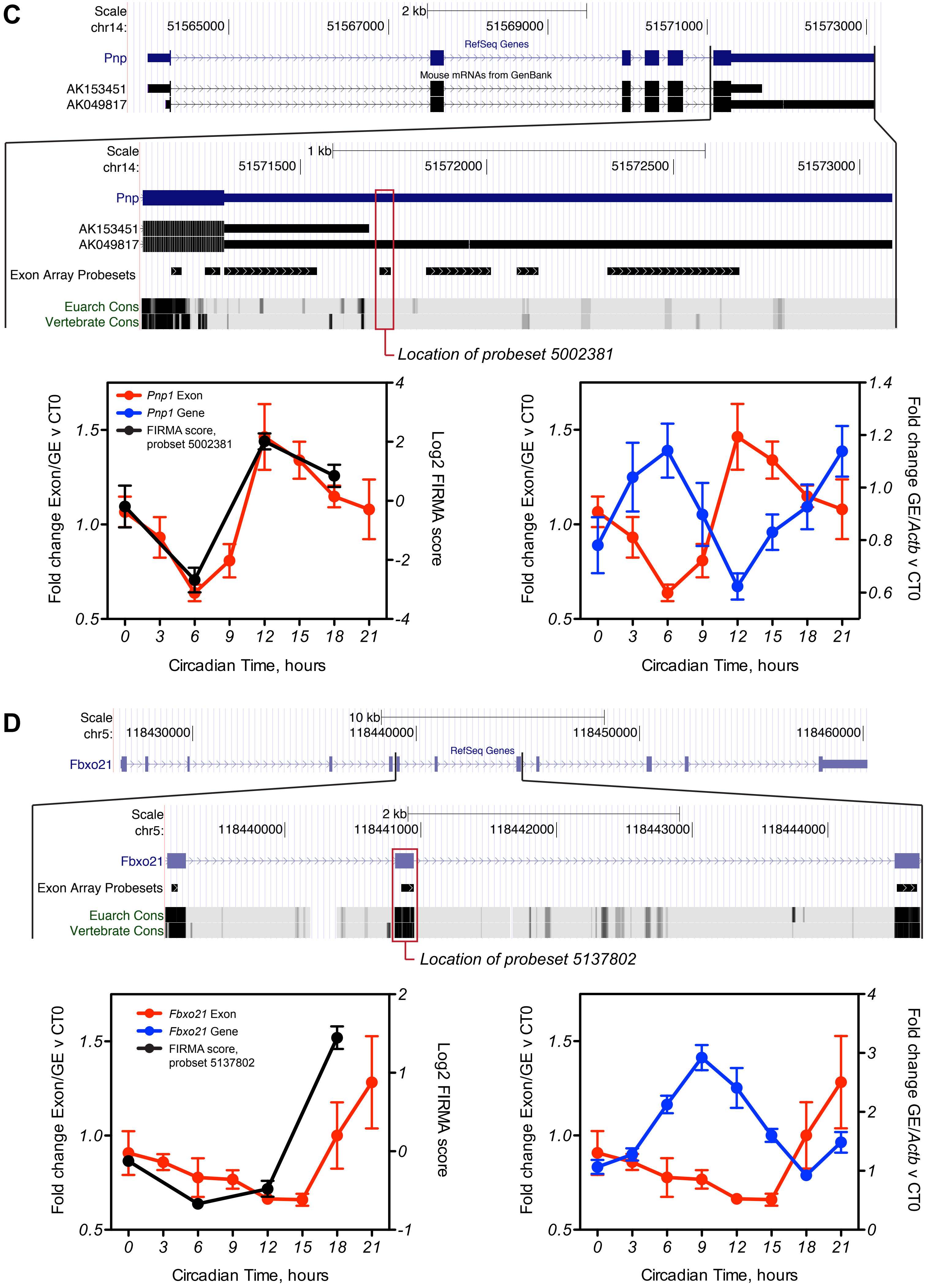

E

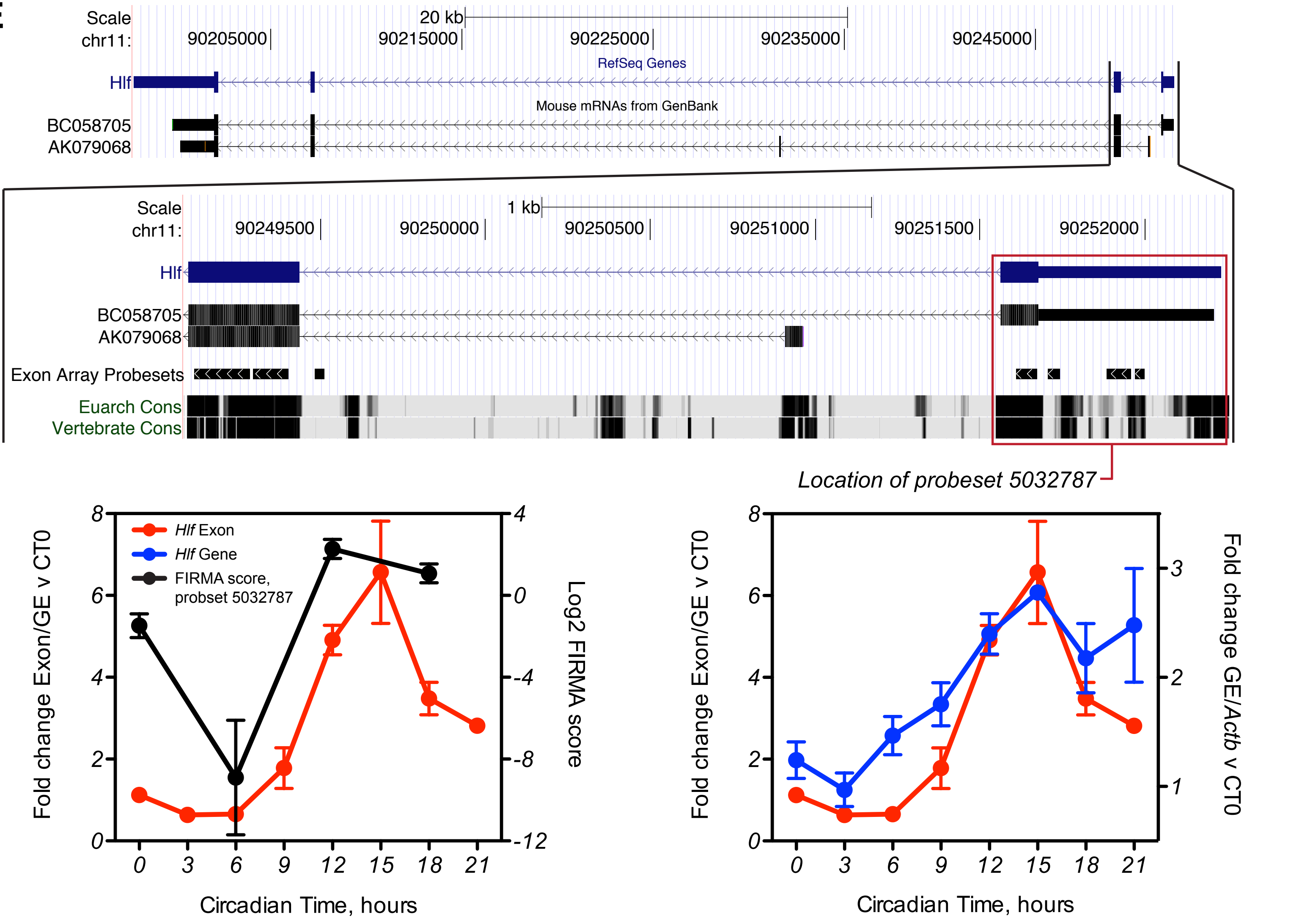
Scale
chr11:
20 kb
90205000
90215000
90225000
90235000
90245000
RefSeq Genes
Hlf
Mouse mRNAs from GenBank
BC058705
AK079068
Scale
chr11:
1 kb
90249500
90250000
90250500
90251000
90251500
90252000
Hlf
BC058705
AK079068
Exon Array Probesets
Euarch Cons
Vertebrate Cons
Location of probeset 5032787
Hlf Exon
Hlf Gene
FIRMA score, probset 5032787
Fold change Exon/GE v CT0
Log2 FIRMA score
Circadian Time, hours
Fold change GE/Actb v CT0
Circadian Time, hours

F

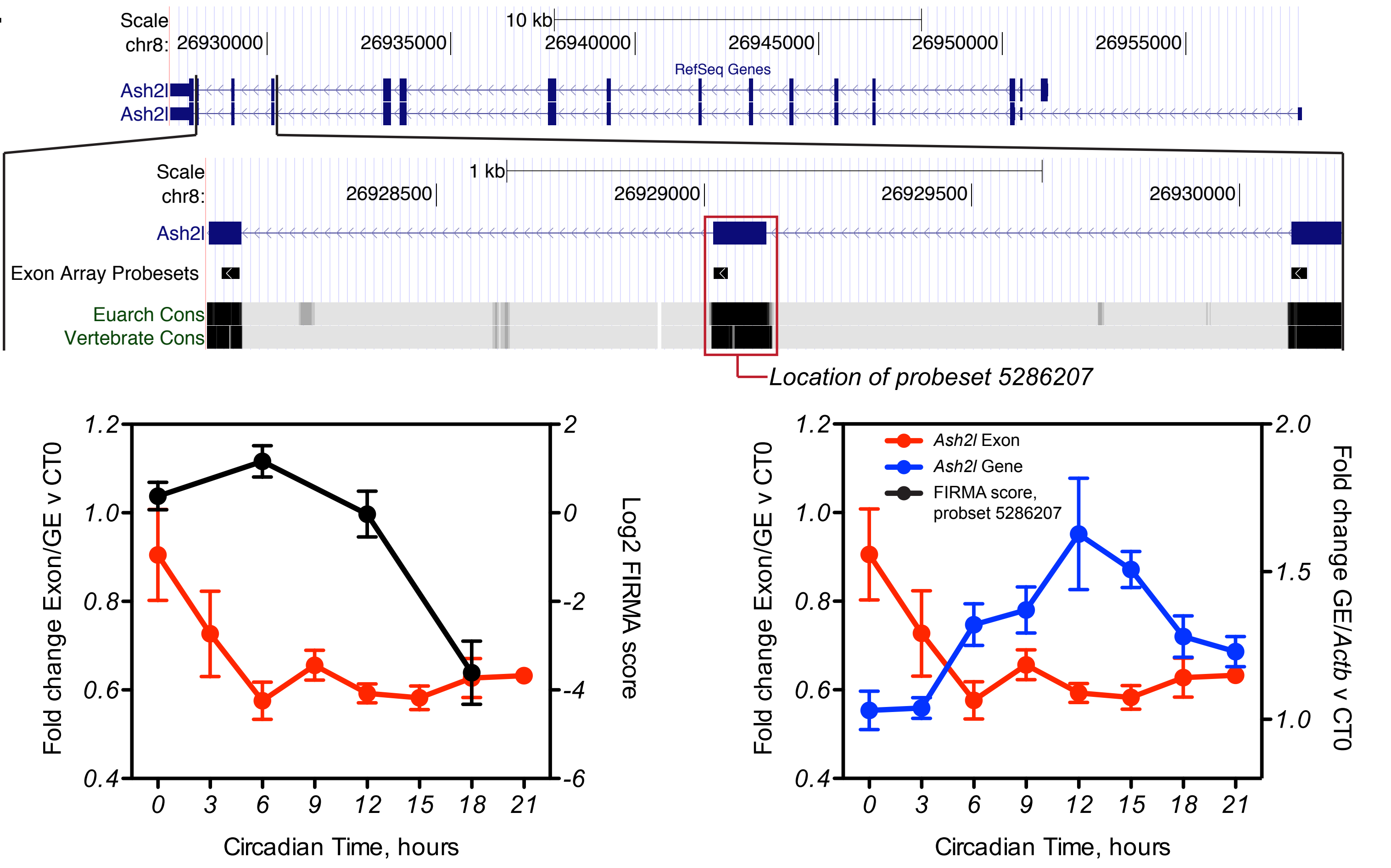
Scale
chr8:
10 kb
26930000
26935000
26940000
26945000
26950000
26955000
RefSeq Genes
Ash2l
Ash2l
Scale
chr8:
1 kb
26928500
26929000
26929500
26930000
Ash2l
Exon Array Probesets
Euarch Cons
Vertebrate Cons
Location of probeset 5286207
Fold change Exon/GE v CT0
Log2 FIRMA score
Circadian Time, hours
Ash2l Exon
Ash2l Gene
FIRMA score, probset 5286207
Fold change GE/Actb v CT0
Circadian Time, hours

mcglincy_figS3iv

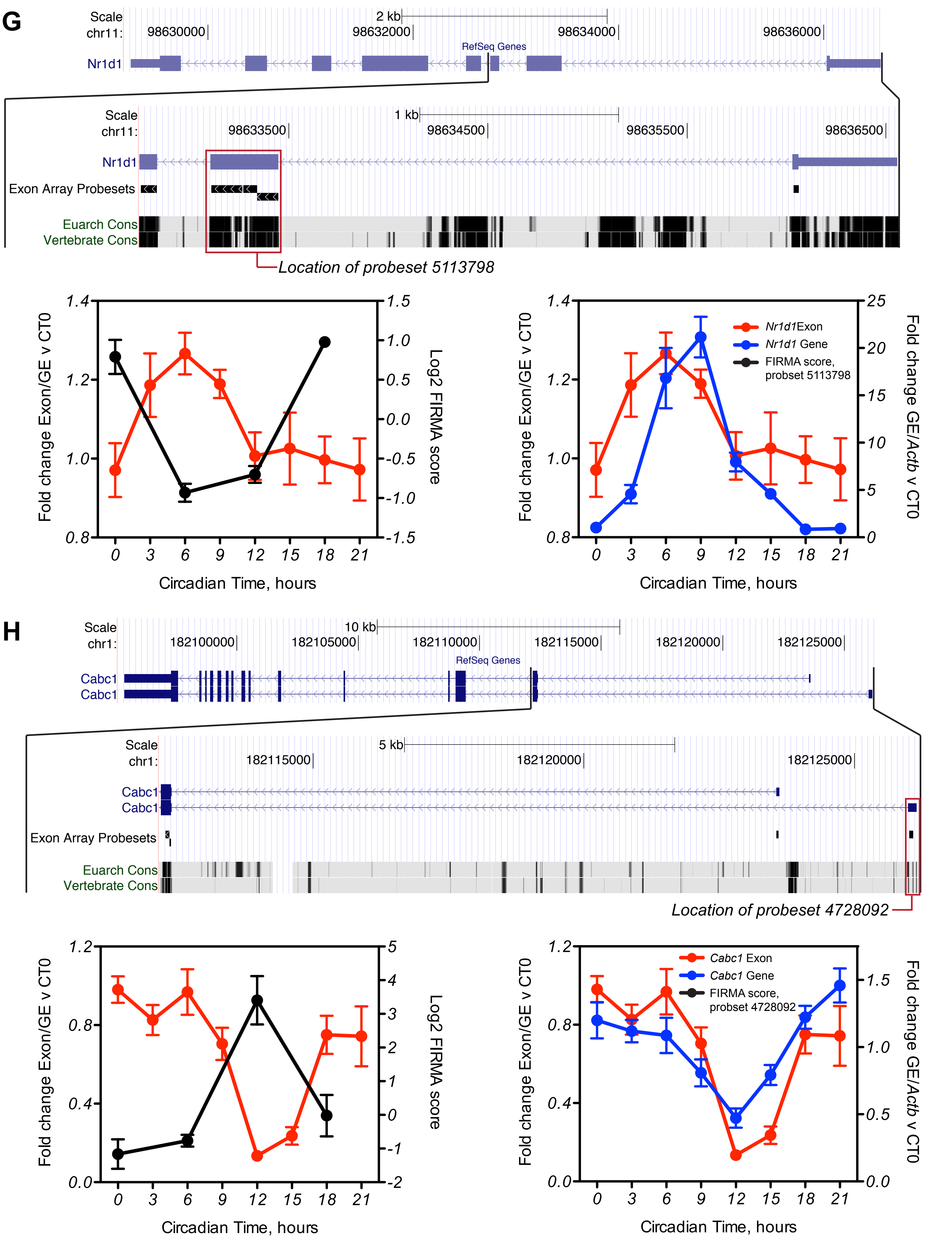

I

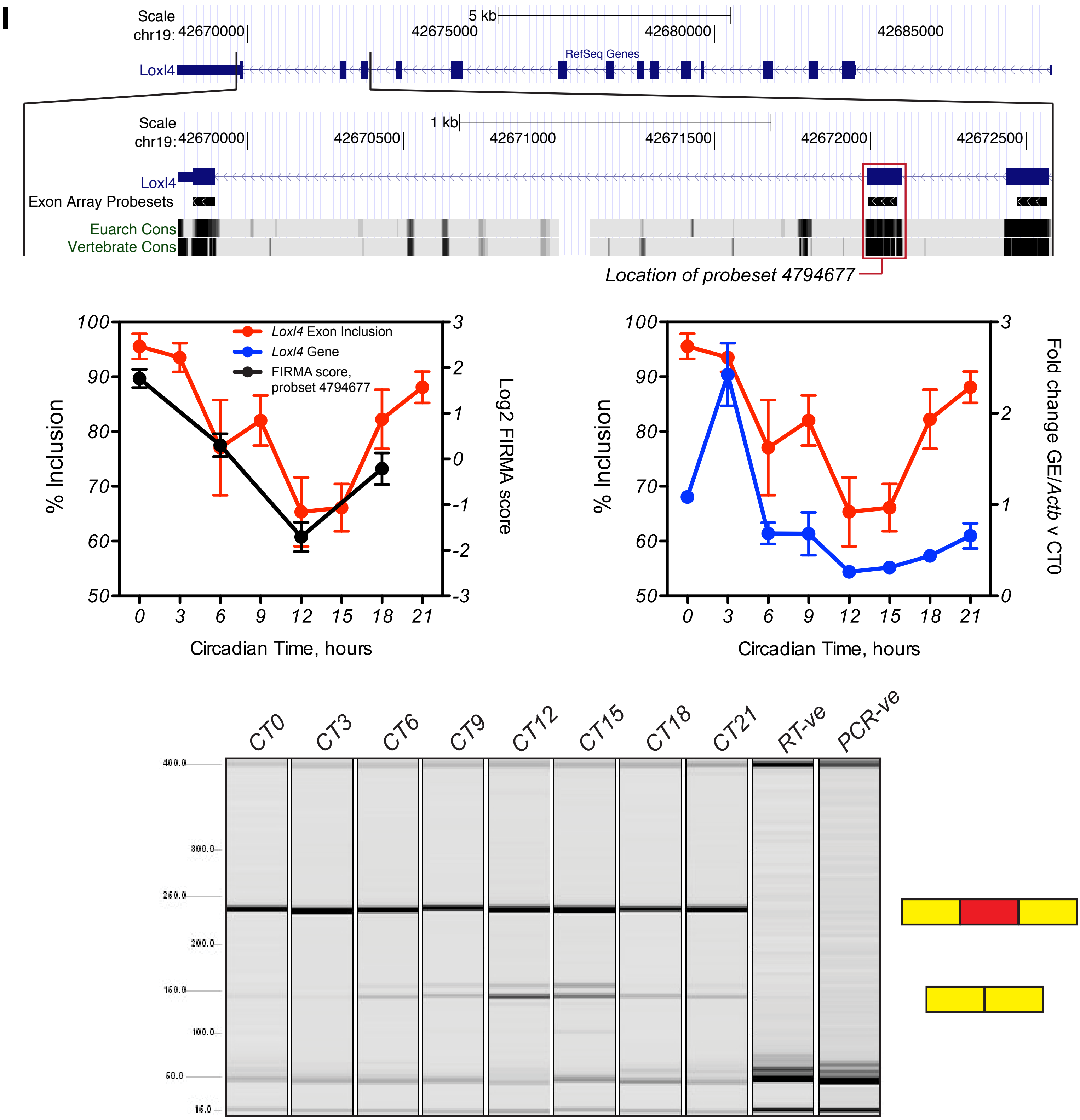
Scale
chr19:
5 kb
42670000
42675000
42680000
42685000
RefSeq Genes
Loxl4
Scale
chr19:
1 kb
42670000
42670500
42671000
42671500
42672000
42672500
Loxl4
Exon Array Probesets
Euarch Cons
Vertebrate Cons
Location of probeset 4794677
% Inclusion
Loxl4 Exon Inclusion
Loxl4 Gene
FIRMA score, probset 4794677
Log2 FIRMA score
Circadian Time, hours
Fold change GE/Actb v CT0
CT0
CT3
CT6
CT9
CT12
CT15
CT18
CT21
RT-ve
PCR-ve

mcglincy_figS3vi

J

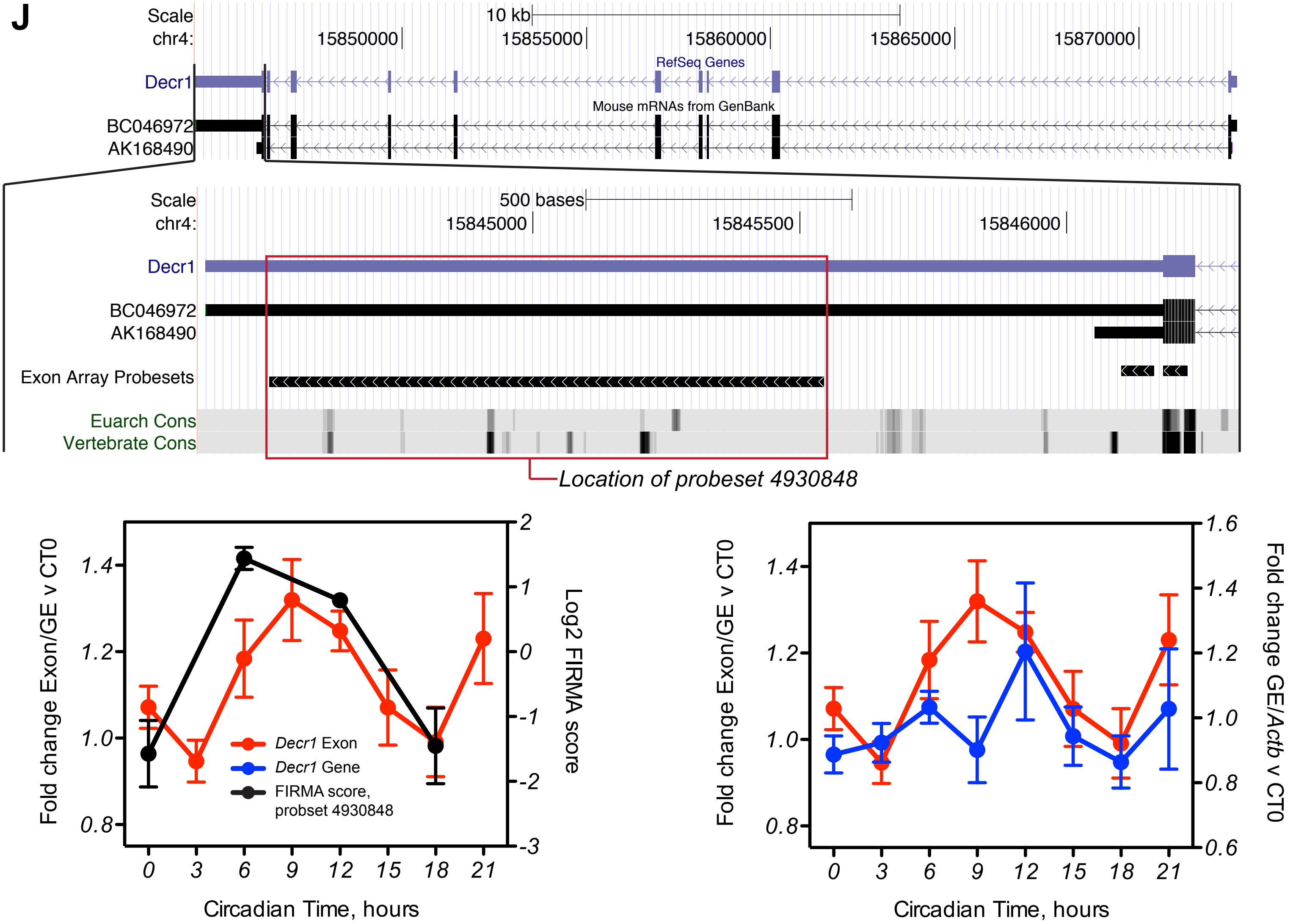

K

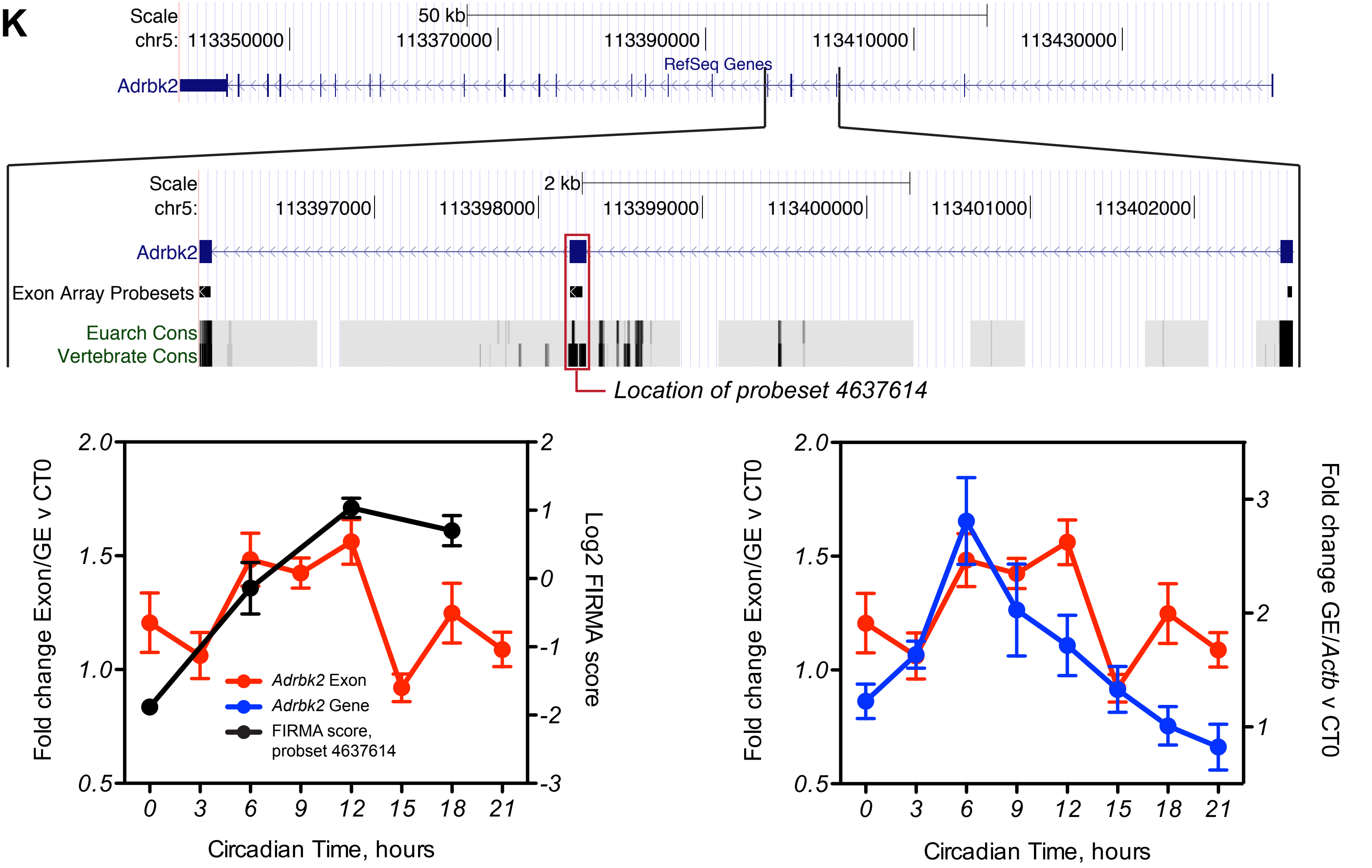

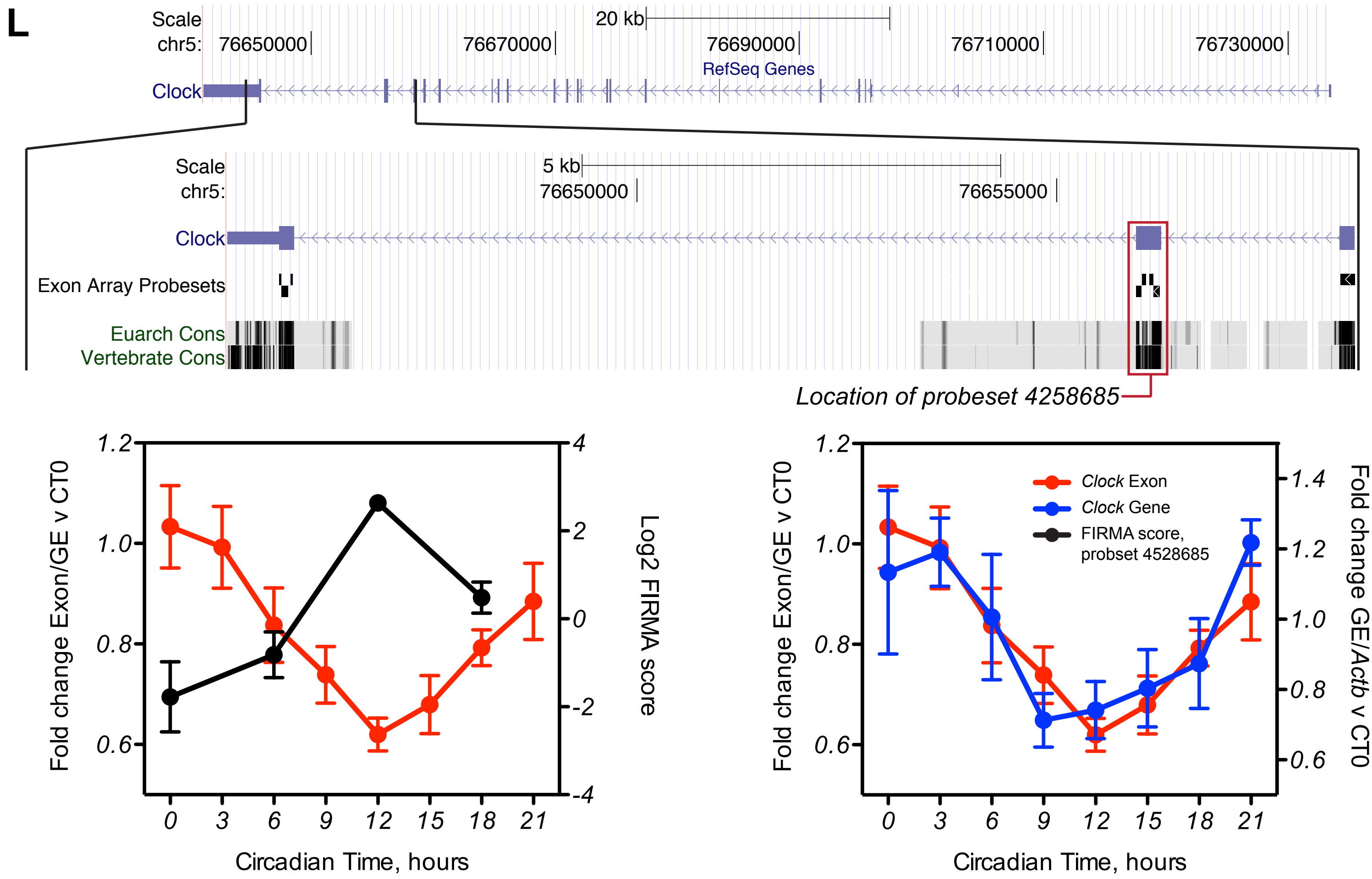
L
Scale
chr5:
20 kb
76650000
76670000
76690000
76710000
76730000
RefSeq Genes
Clock
Scale
chr5:
5 kb
76650000
76655000
Clock
Exon Array Probesets
Euarch Cons
Vertebrate Cons
Location of probeset 4258685
Fold change Exon/GE v CT0
Log2 FIRMA score
Circadian Time, hours
Clock Exon
Clock Gene
FIRMA score, probeset 4528685
Fold change GE/Actb v CT0
Circadian Time, hours

mcglincy_figS4i

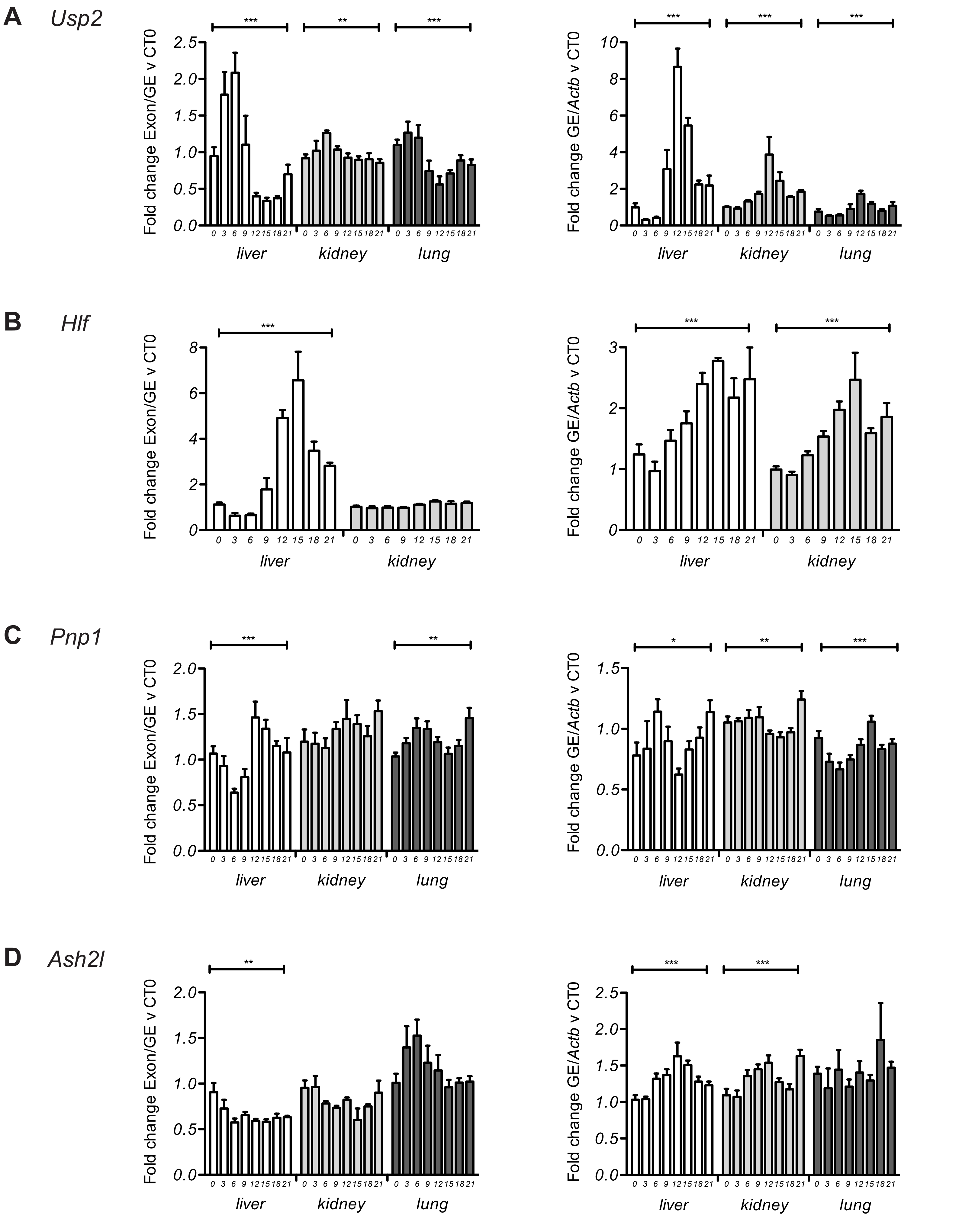

mcglincy_figS4ii

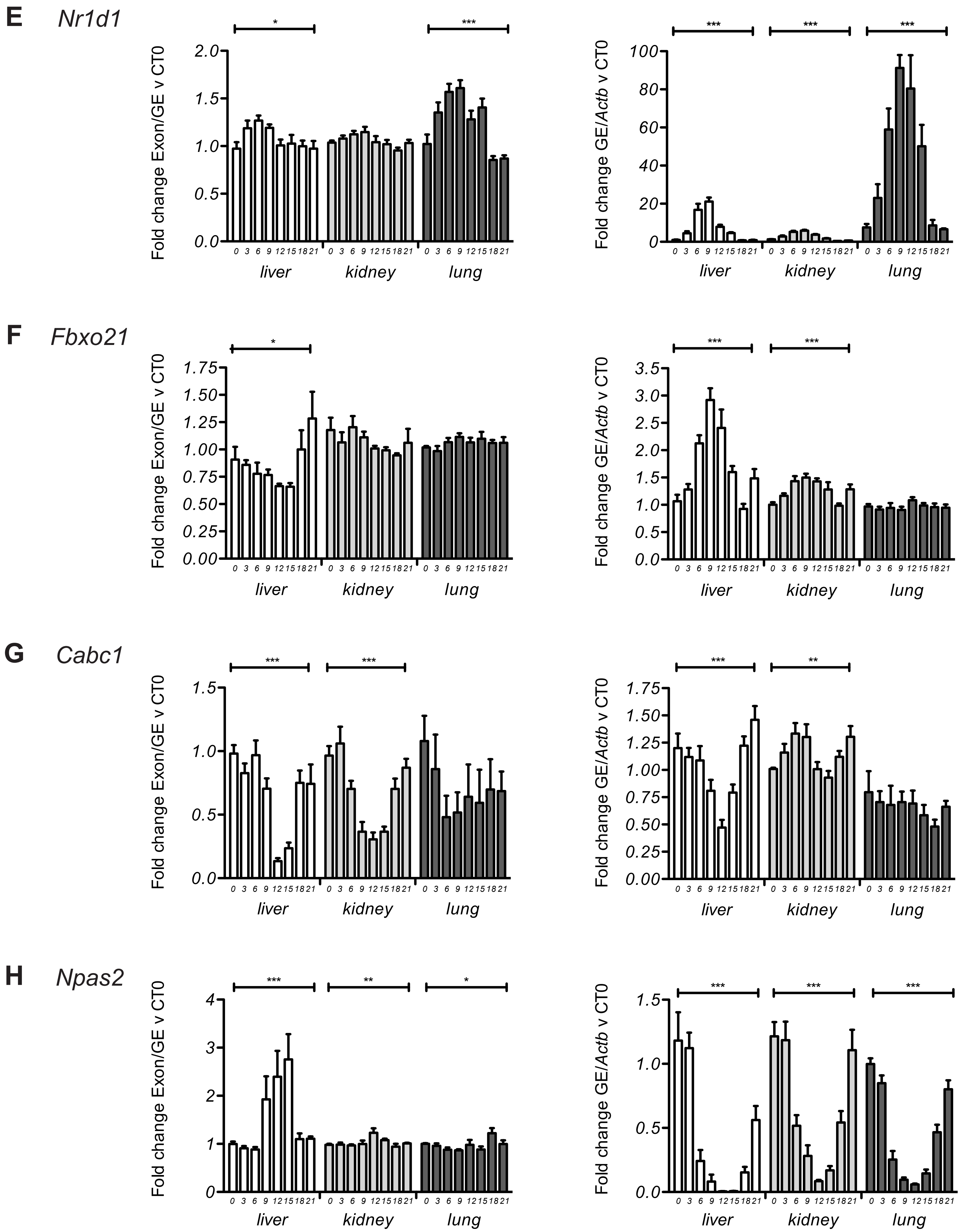

mcglincy_figS5

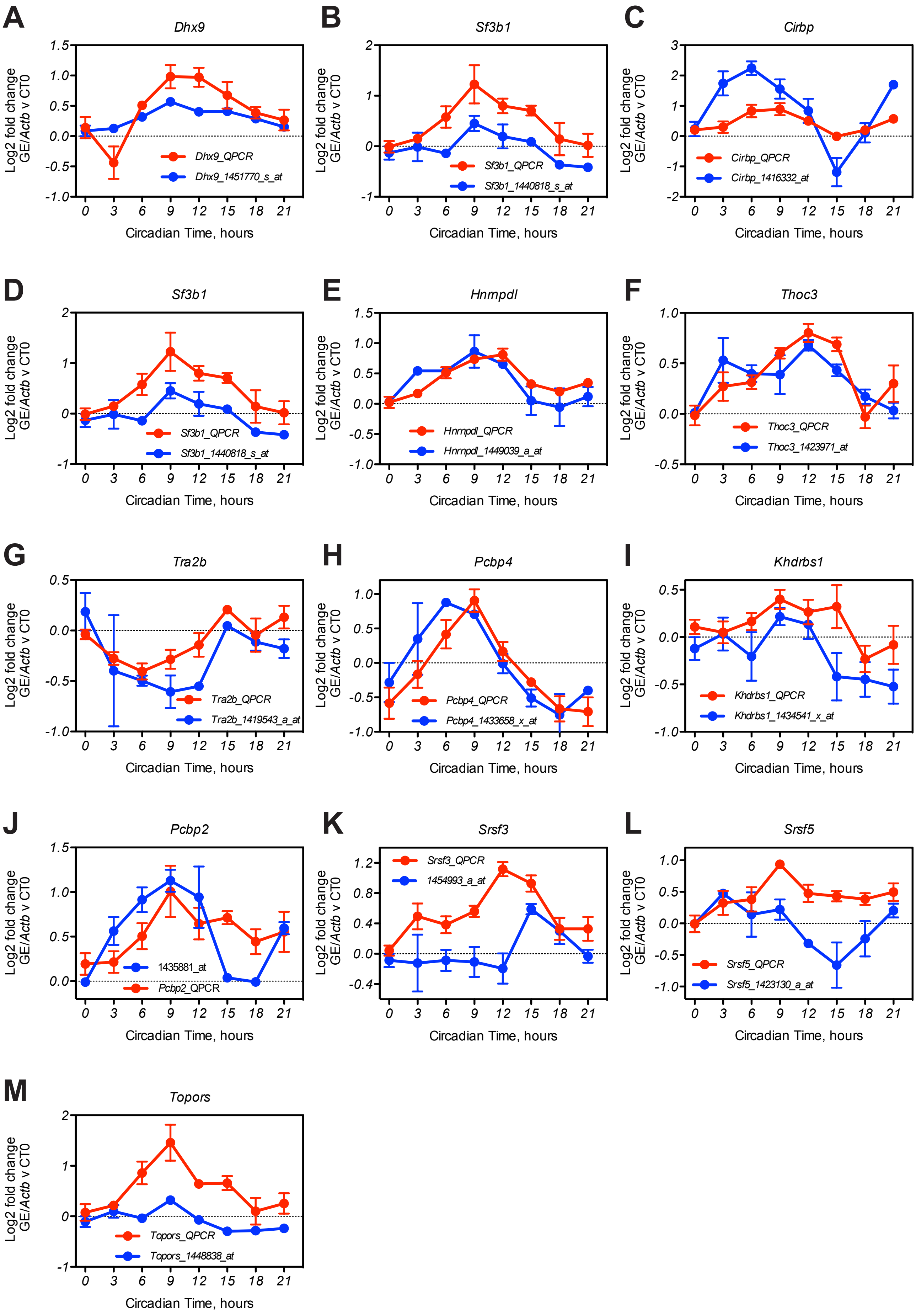

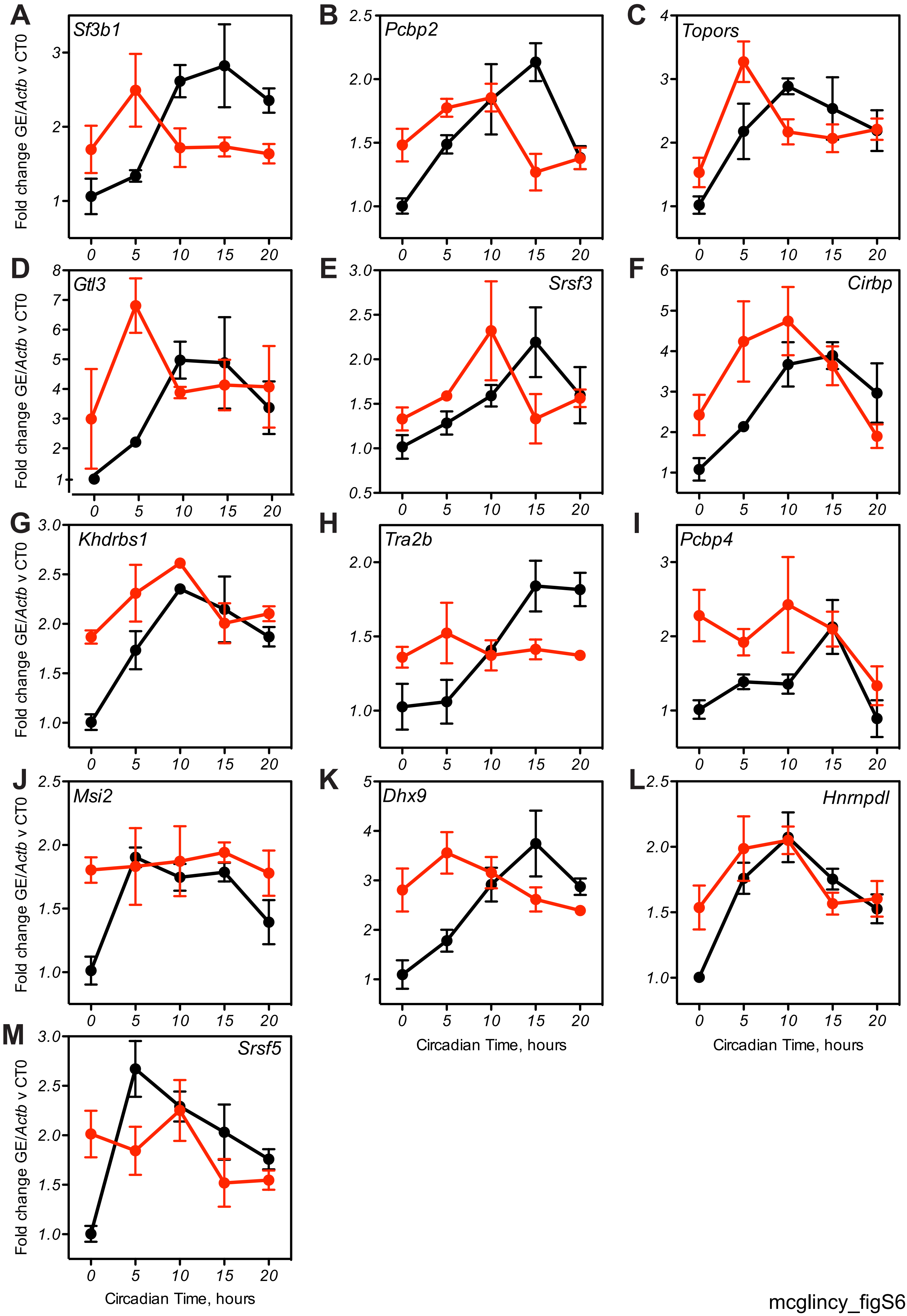

mcglincy_figS6

A

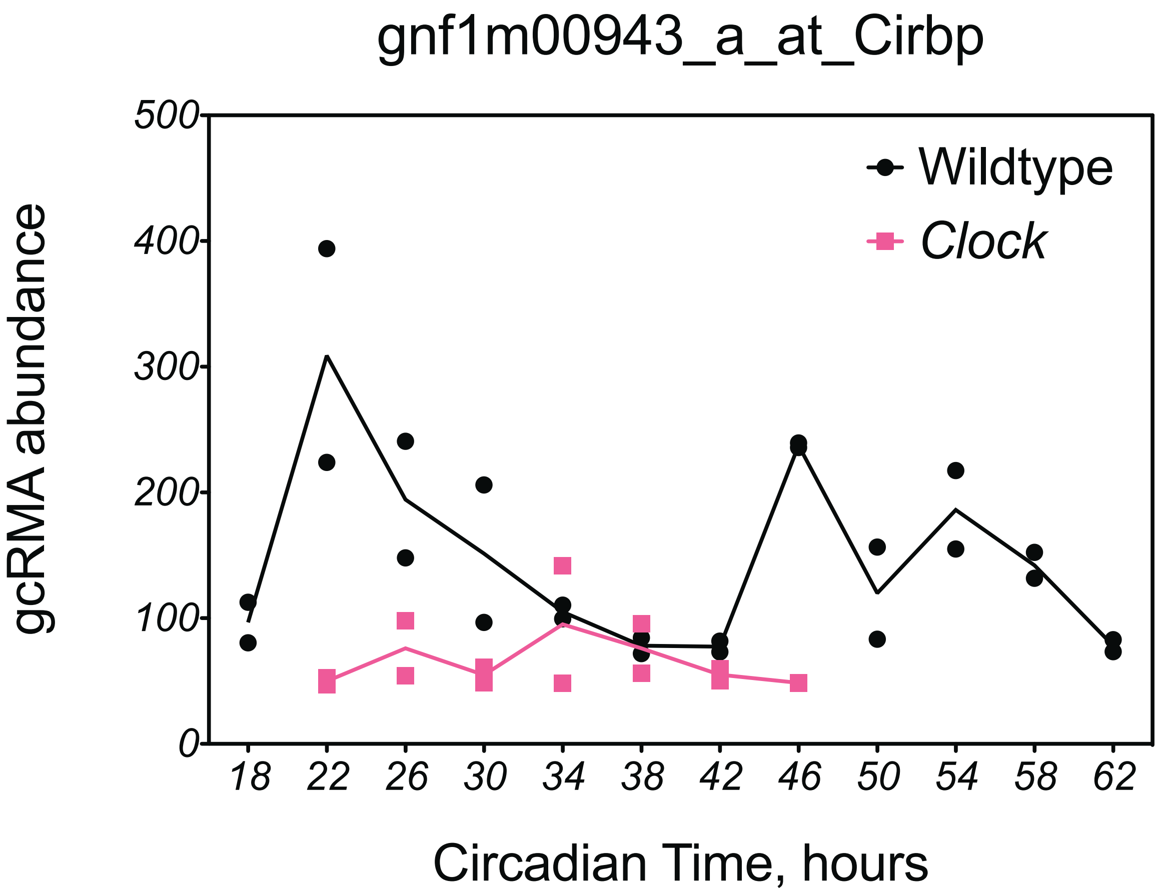

B

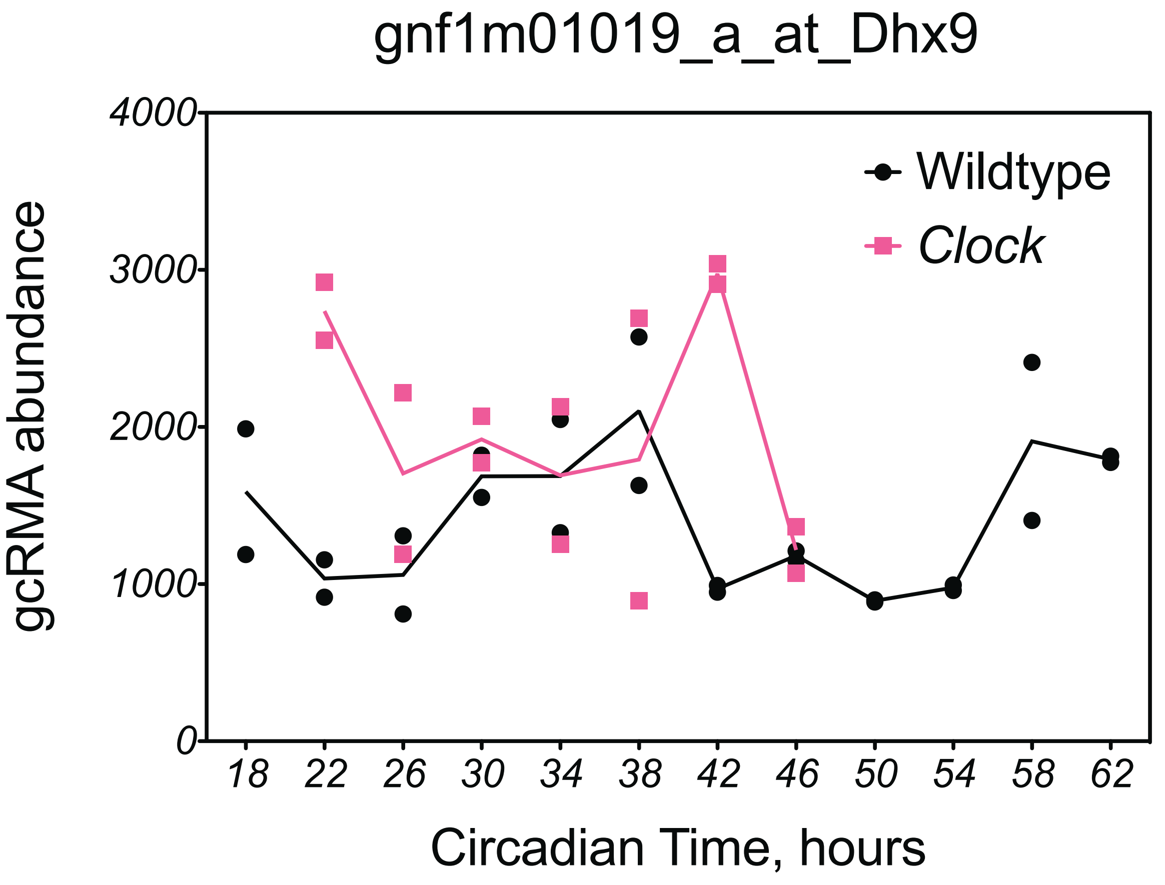

mcglincy_figS7

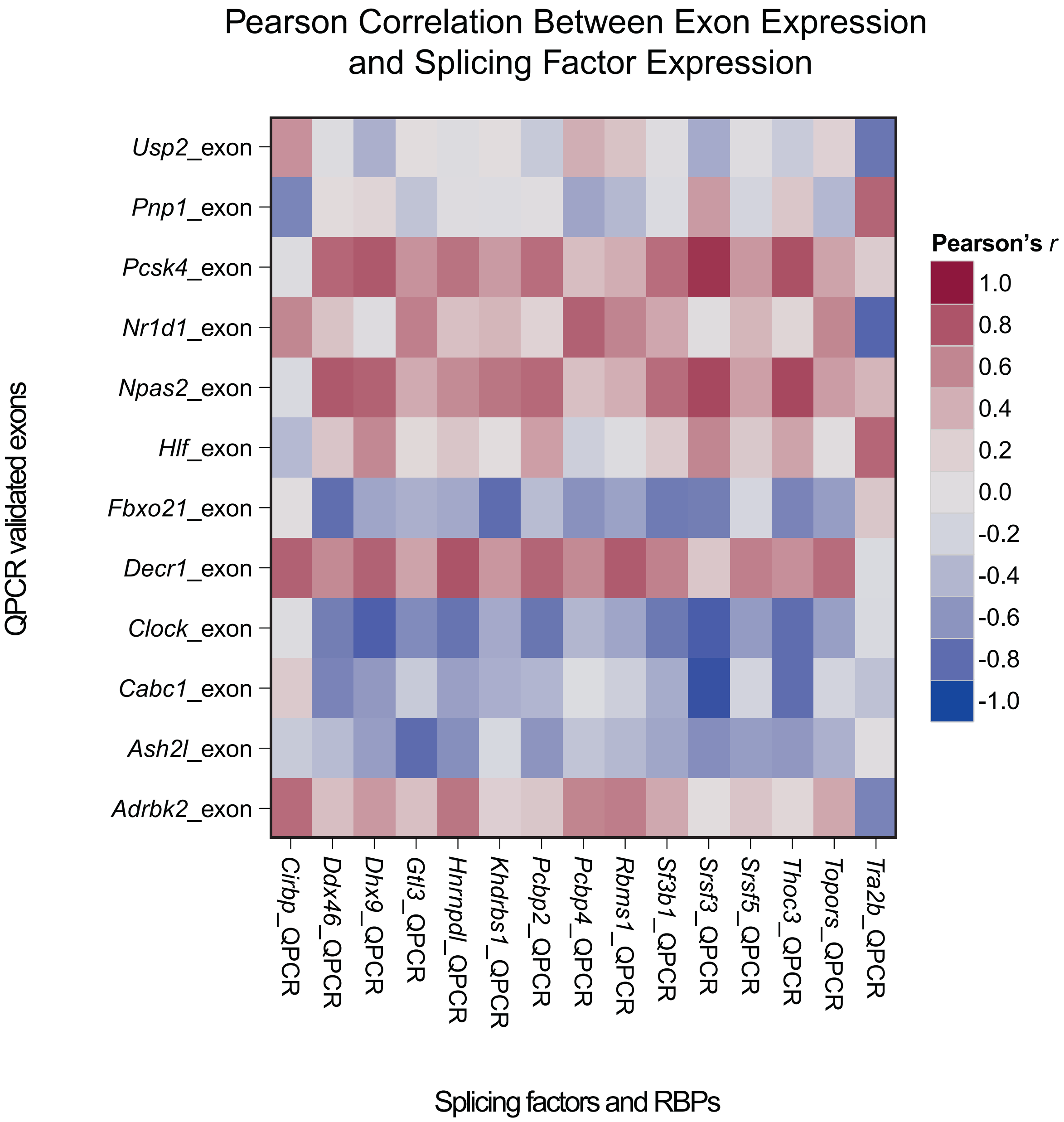

mcglincy_figS8

A

B

C

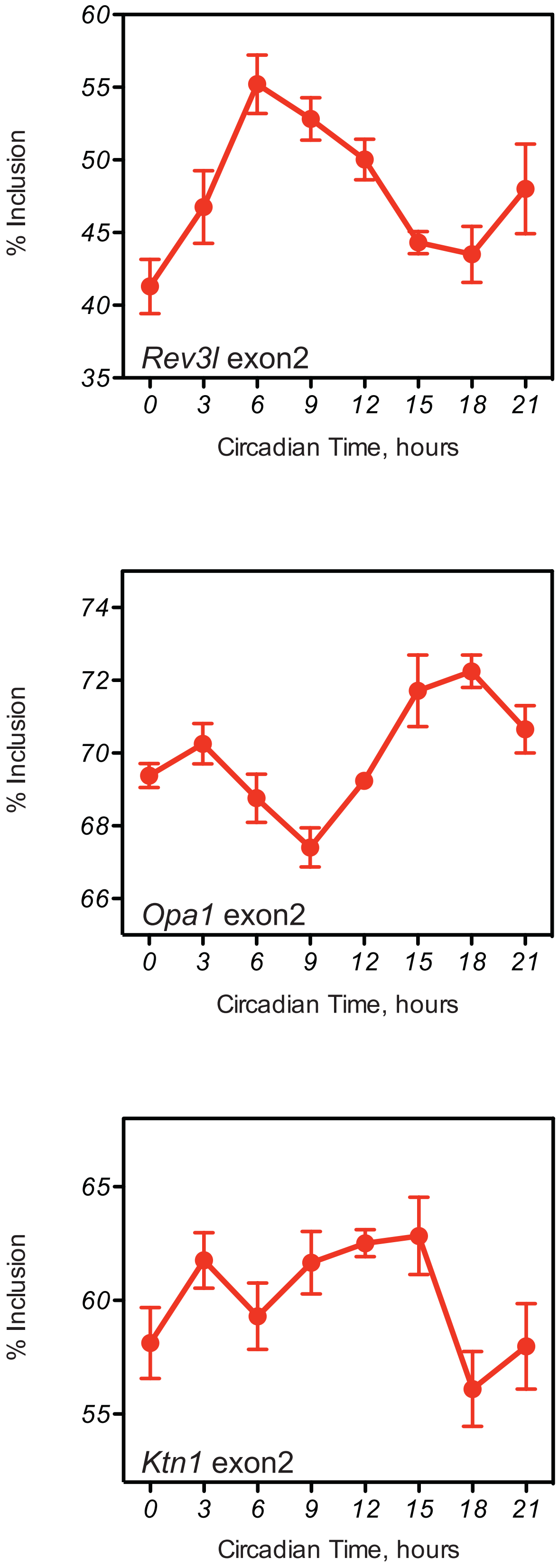

mcglincy_figS9

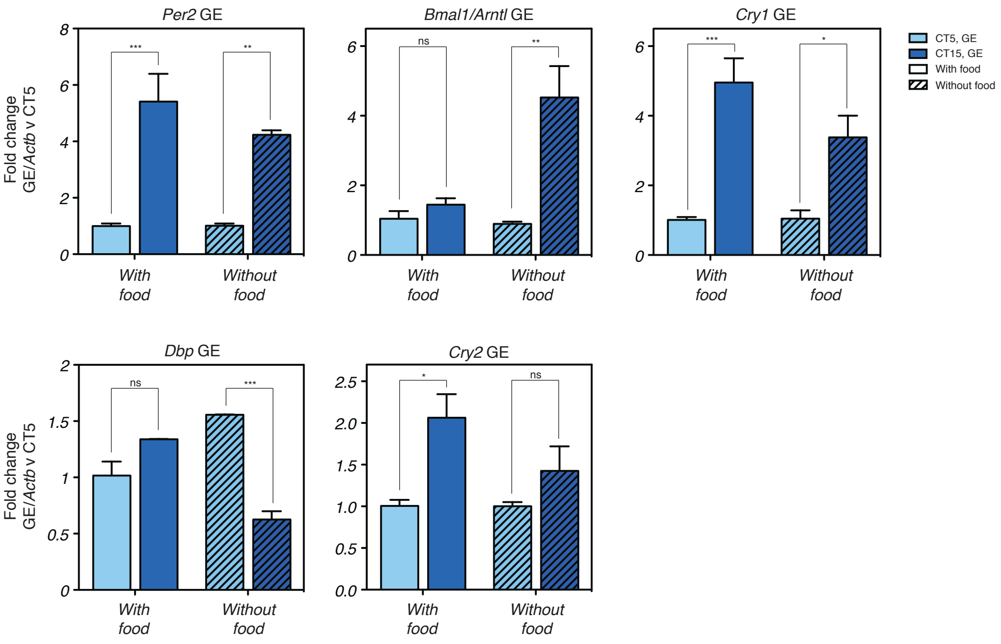

mcglincy_figS10
